# The validity and reliability of the Malay version of the social support for exercise and physical environment for physical activity scales

**DOI:** 10.1371/journal.pone.0239725

**Published:** 2020-09-28

**Authors:** Abdulwali Sabo, Yee Cheng Kueh, Wan Nor Arifin, YoungHo Kim, Garry Kuan

**Affiliations:** 1 Biostatistics and Research Methodology Unit, School of Medical Sciences, Universiti Sains Malaysia, Kubang Kerian, Kelantan, Malaysia; 2 Department of Sports and Health Science, Seoul National University of Science and Technology, Seoul, South Korea; 3 Exercise and Sports Science, School of Health Sciences, Universiti Sains Malaysia, Kubang Kerian, Kelantan, Malaysia; University of Birmingham, UNITED KINGDOM

## Abstract

**Background:**

This study aimed to determine the validity of the Malay-translated version scales for assessing the social support and physical environment for exercise activities.

**Method:**

The study was a cross-sectional design in nature, using self-reported questionnaires among the university students in Malaysia. Participants were selected using a convenience sampling approach. Perceptions regarding social support and physical environment were assessed using the Malay-translated version scales. The standard forward-backwards translation was conducted to translate the English version of the scales to the Malay version. Confirmatory factor analysis (CFA) was used to validate the translated version scales; composite reliability (CR) and average variance extracted (AVE) were computed.

**Results:**

A total of 857 students participated in this study (female: 49.1%, male: 50.9%). The mean age of the participants was 20.2 (SD = 1.6). The fit indices of the initial hypothesized measurement models (social support and physical environment) were not satisfactory. Further improvements were made by adding covariances between residuals' items within the same factor for each hypothesized model. The final re-specified measurement models demonstrated adequate factor structure for the social support scale with 24 items (CFI = .932, TLI = .920, SRMR = .054, RMSEA = .061), and the physical environment scale with five items (CFI = .994, TLI = .981, SRMR = .013, RMSEA = .054). The CR was .918 for family support, .919 for friend support, .813 for perceived availability, and .771 for perceived quality. The AVEs were .560 for family support, .547 for friend support, .554 for perceived availability, and .628 for perceived quality. The intra-class correlation (ICC) based on test-retest was .920 for family support, .984 for friend support, .895 for availability of facilities, and .774 for quality of facilities.

**Conclusion:**

The Malay version of the social support scale for exercise and the physical environment scale for physical activity were shown to have adequate psychometric properties for assessing perceived social support and physical environment among the university students in Malaysia.

**Perspective:**

This study presented the psychometric properties of the social support and physical environment scales based on CFA and was the first to translate these scales from the original English version to the Malay version.

## Introduction

Physical activity is considered one of the essential components that promote healthy living [1]. Participation in regular physical activity can lead to the prevention and treatment of several chronic diseases such as diabetes, cardiovascular disease, obesity, and cancer [2]. According to Nicklett et al. [3], physically inactive adults were 50 to 60% more likely to die early than physically active adults. Also, some studies reported that about 36% of Malaysian adults were physically inactive [4, 5]. Based on global estimates, physical inactivity accounted for 9% of deaths in 2008 [6], whereas sedentary behavior accounted for 3.8% of deaths from 2002 to 2011 [7]. These were reported to be major risk factors that promote noncommunicable diseases [8].

The social ecological model created by Bronfenbrennner [9] has been the most employed model by researchers to illustrate and identify factors associated with physical activity participation [10, 11]. According to this model, the social environment (e.g., family support, friends support, social norms, and cultural background) along with the physical environment (e.g., perceived availability and quality of exercise facilities) plays a vital role in influencing and maintaining physical activity participation [2, 8]. These environmental factors surround the innermost layer of individual factors, such as demographic factors (e.g., gender, age and level of education) and psychological factors (e.g., self-efficacy and decisional balance) [12].

Perceived social support and perceived physical environment were found to be correlated with mental health-related quality of life, and these relationships were significantly mediated by leisure-time walking and moderate-to-vigorous physical activity [13]. A previous study that was conducted to examine the effectiveness of worksite social and physical environment intervention on the need for recovery, relaxation, and physical activity reported the largest improvement in the combined intervention group [14]. According to Ishii, Shibata, and Oka [15], social support mediated the effect of the physical environmental factors on walking, moderate-intensity activity (excluding walking), and vigorous-intensity activity. Acknowledging the combined contributions of these environmental variables on physical activity behavior, there is a need to have scales that will precisely reflect these factors.

Several notions and classifications have been applied to explain the social environment (SE) support and its constructs [16–18]. Despite SE support having multiple complex dimensions, it represents the aid and assistance shared through social contacts and interpersonal activities [19]. SE support signifies the concept for the availability of supportive actions or perceptions when demanded. This illustrates the relative distinction between the SE support an individual gets and the individual’s perception of such support. While numerous studies fail to establish a precise distinction between perceived and received SE support, perceived SE support is reportedly more strongly associated with positive health outcomes [18, 20].

According to Sallis, Bauman, Pratt [21], the physical environment (PE) is the degree of available compensations or limitations that determine health practice suitability. Incentive environments are those that present the best available exercise facilities, such as sports fields, bicycle paths, and swimming pools. Restricting environments are those that restrain access or promote sedentary environments, for instance, busy highway systems and idle games places [21]. Perceived measures of a PE represent the vital and distinct dimensions that influence physical activity participation and allow for evaluating important aspects of the PE that are hard to assess objectively, such as safety of environment and aesthetics [22].

Social support scale for exercise was first originated by Sallis, Grossman, Pinski, Patterson, and Nader [23] and was confirmed as a valid and reliable instrument among 171 subjects with a mean age of 21.3 years (SD = 6.5). The physical environment scale for physical activity was first originated by Stahl et al. [24] and was confirmed as a valid and reliable instrument among 3343 adults, ages 18 years and above. These two scales were translated into the Korean language and confirmed to be valid and reliable measures for assessing participants’ perceptions regarding social support for exercise and available physical environment for physical activity [25]. The validity and reliability of these translated versions were supported by other researchers [11, 26]. The social support and physical environment scales have been translated into and verified in other languages, including Chinese [27] and Japanese [15]. However, there are not yet available Malay versions of these scales.

Given that, the university students are mostly adults, it is necessary to authenticate valid measurement scales for assessing their perceived social support and physical environment support for participation in physical activity. The previous study by Bostock. [28] reported that parents play a vital role in facilitating young children’s physical activity. Also, parents are more supportive of physical activity when there is a safe and available physical environment [29]. It is therefore necessary to identify the components that support and sustain physical activity participation. The present study aimed to translate the social support and physical environment scales for exercise into Malay for application among Malay university student’s populations and to confirm the psychometric properties of the social support and physical environment scales.

## Method

### Participants

A total of 857 undergraduate students (male: 436, female: 421, M_age_ = 20.2 years, SD = 1.6) were recruited through a convenience sampling approach. Larger samples generally produce robust results with greater precision [30]. Therefore, in this study, a sample size of 857 is adequate for confirmatory factor analysis (CFA) of the 24-item social support scale and the 5-item physical environment scale. The students identified themselves as Malay (81.2%), Chinese (11.4%), Indian (4.4%), and other (3.0%) but were all Malaysians with profound reading and speaking skills in Malay. Majority of the students (80.6%) had a mean of 1.77 days per week for physical activity participation and a mean of 62.3 minutes per session. The participants' reported sports activities were football, badminton, basketball, jogging, cycling, tennis, and netball.

### Questionnaire translation

The English versions of the scales derived from previous studies were translated into the Malay language by employing the following steps. First, a bilingual researcher familiar with the scales translated the English versions of the social support scale for exercise and physical environment scale for physical activity scales into the Malay language, as well as maintaining the content meaning of the scales. Second, the translated Malay version was back-translated into English by a native Malay speaker who also speaks English. Third, these two versions were reexamined and settled by a panel of five experts in health psychology, sport sciences, physical education, and sports psychology. The panel members were all native Malay speakers who also speak English and had more than 10 years of work experience in their fields of expertise. The panel evaluated the versions, comparing each item to its corresponding item in the original English version. All discrepancies were properly amended. The items were further appraised by the panel to ascertain whether they were culturally fit for Malaysian populations.

The researchers invited 10 undergraduate students to evaluate the clarity of the final Malay versions. They were asked to give their views of each item on the questionnaire’s content and display. Their comments were consistent and required no amendments. The Malay-translated versions of the social support scale for exercise and the physical environment scale for physical activity are presented in the S1 Appendix.

### Data collection

The researchers obtained approval from the Human Research Ethics Committee of Universiti Sains Malaysia and the study was conducted according to the Declaration of Helsinki. The data collection was between September and December 2018 at Universiti Sains Malaysia (USM), located in Kubang Kerian, Kelantan, Malaysia. The study was a cross-sectional design using the self-reported social support scale and the physical environment scale. Researchers reached out to the lecturers before beginning their classes to brief them about data collection and after the classes, the students who agreed to take part in the study remained behind. The information sheets on participation were distributed to the students to read before being asked to complete the questionnaire. Implied consent was obtained when the participants volunteered to complete both the social support and the physical environment questionnaires and returned them to the researchers. The average time spent to complete both questionnaires was between 20 and 27 minutes.

A total of 918 questionnaires were returned to the researchers, and 857 had complete responses to all the items of social support scale and physical environment scale. Hence, the final sample was 857 without missing values. To assess the test-retest reliability of the social support and physical environment scales, a total of 120 participants again completed and returned the questionnaires at day 14.

### Measures

#### Demographic and sports activities information

Other items included in the questionnaire are the participants’ demographic characteristics (e.g., age, gender, and ethnicity), physical activity levels, sports participation, and the hours spent performing physical activity or sports per week.

#### Social support scale for exercise

The social support scale as originated by Sallis et al. [23] had 20 items with the family support for exercise scale (two factors, 15 items) extracted with 46.2% of total variance explained and friend support for exercise scale (one factor, five items) extracted with 35% of the total variance explained. These scales were revised and translated into Korean [25]. The revised scale consisted of 24 items (i.e., 12 reflecting family support and 12 reflecting friend support). Participants rated their perceived support, such as “I have a friend or acquaintance who encouraged me to exercise,” with five-point response options ranging between 1 (never) and 5 (very often) [11]. Also, a two-week, test-retest reliability coefficient was reported as .83 for family support and .89 for friends support [25]. In this study, the Malay version of the social support scale for exercise was based on the revised version by Yang et al. [25].

#### Physical environment scale for physical activity

The physical environment was first originated by Ståhl et al. [24] with three items measuring perceived local opportunities to be physically active, together with two additional items, one item measuring general awareness of opportunities, and one item measuring the contribution of health policy to promote physical activity. The physical environment scale was revised and translated into Korean [25]. The revised version consisted of five items: three items assessing availability and access to physical activity facilities and two items assessing the quality of physical activity facilities. The study participants rated their perceptions of each statement, such as “my residential area offers enough facilities for me to be physically active”, with five-point response options ranging between 1 (not true at all) and 5 (definitely true) [11]. Also, a two-week, test-retest reliability coefficient was reported as .91 [25]. In this study, the Malay version of the physical environment scale for physical activity was based on the revised version by Yang et al. [25].

### Statistical analysis

The data were pre-screened to check for wrong data entry and missing values, and only the questionnaires with complete responses were included in the analysis. CFA analysis was conducted using Mplus 8 to test the initial hypothesized models. In this study, the MLR Estimator was preferred to perform CFA because of its robustness to non-normal data distributions and because it provides robust estimates with standard errors, including a mean adjusted chi-square statistic [31].

The two initial measurement models of social support with 24 items and physical environment with five items were tested using CFA. The standardized factor loading of .40 or greater was applied as a cut-off to establish sufficient factor loading for all the items, as such, employed as a criterion to retain or remove an item [32, 33]. According to Hair et al. [34], the recommended fit indices for a sample size larger than 250 with more than 12 items for social support and less than or equal to 12 items for the physical environment were: root mean square error of approximation (RMSEA) with a desired value of less than .07; standardized root mean square residual (SRMR) with a desired value of less than .08; and comparative fit index (CFI) or Tucker and Lewis index (TLI) with the desired values of more than .92 and .97 for social support and physical environment respectively [34]. Model re-specification was conducted by referring to the CFA modification index to improve the model fit indices. The models were re-specified after the researchers considered sufficient theoretical guide.

In CFA, convergent validity and discriminant validity were the components for assessing construct validity among item measures. The convergent validity refers to the amount of variance shared by items of a specific construct [34]. According to Hair et al. [34], the factor loadings, AVE, and reliability were among the several ways available for estimating the relative amount of convergent validity. For reliability, Cronbach's alpha remains the commonly reported coefficient in the literature [34], however, composite reliability (CR) is advocated because Cronbach's alpha may underestimate reliability and CR provides a better estimate when residual covariances were included in the model [35]. In this study, because residual covariances were added for both the social support and physical environment models and previous studies [23–25, 27] reported only Cronbach's alpha, we reported the CR and the Cronbach's alpha coefficient. CR was estimated using Raykov’s method [35] in Mplus 8. The cutoff values were greater than or equal to .60 for CR [36] and .50 for AVE [37].

Discriminant validity, or the extent to which a factor is distinct from other factors [38], was examined by investigating the correlations between the factors in the models. A correlation coefficient of .85 or less between factors was considered adequate discriminant validity [39]. Additionally, for discriminant validity to be supported, Fornell and Larcker [37] noted that the AVE of the constructs needs to be higher than the shared amount of variance (i.e., square of the correlation) between the constructs. For test re-test reliability, a sub-sample of 120 participant’s scores was used to determine the intra-class correlation (ICC) for the social support and physical environment scales. Sufficient reliability was attained when the ICC values were greater than .70 [40]. SPSS 24 was used to compute the Cronbach's alpha and ICC values.

## Results

### Social support for exercise measurement model

**Fig 1:**
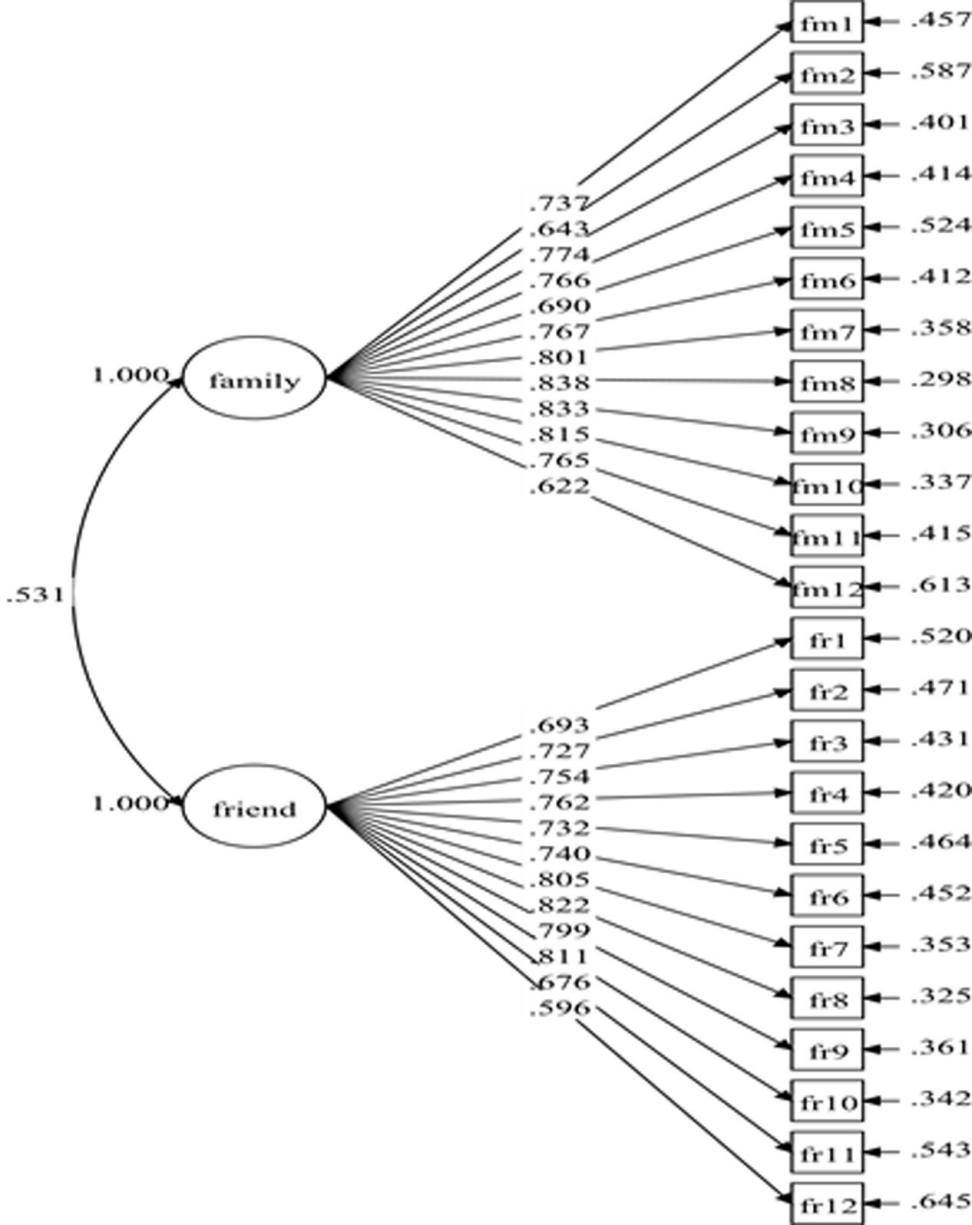
Social support measurement model (Model-1).

**Fig 2:**
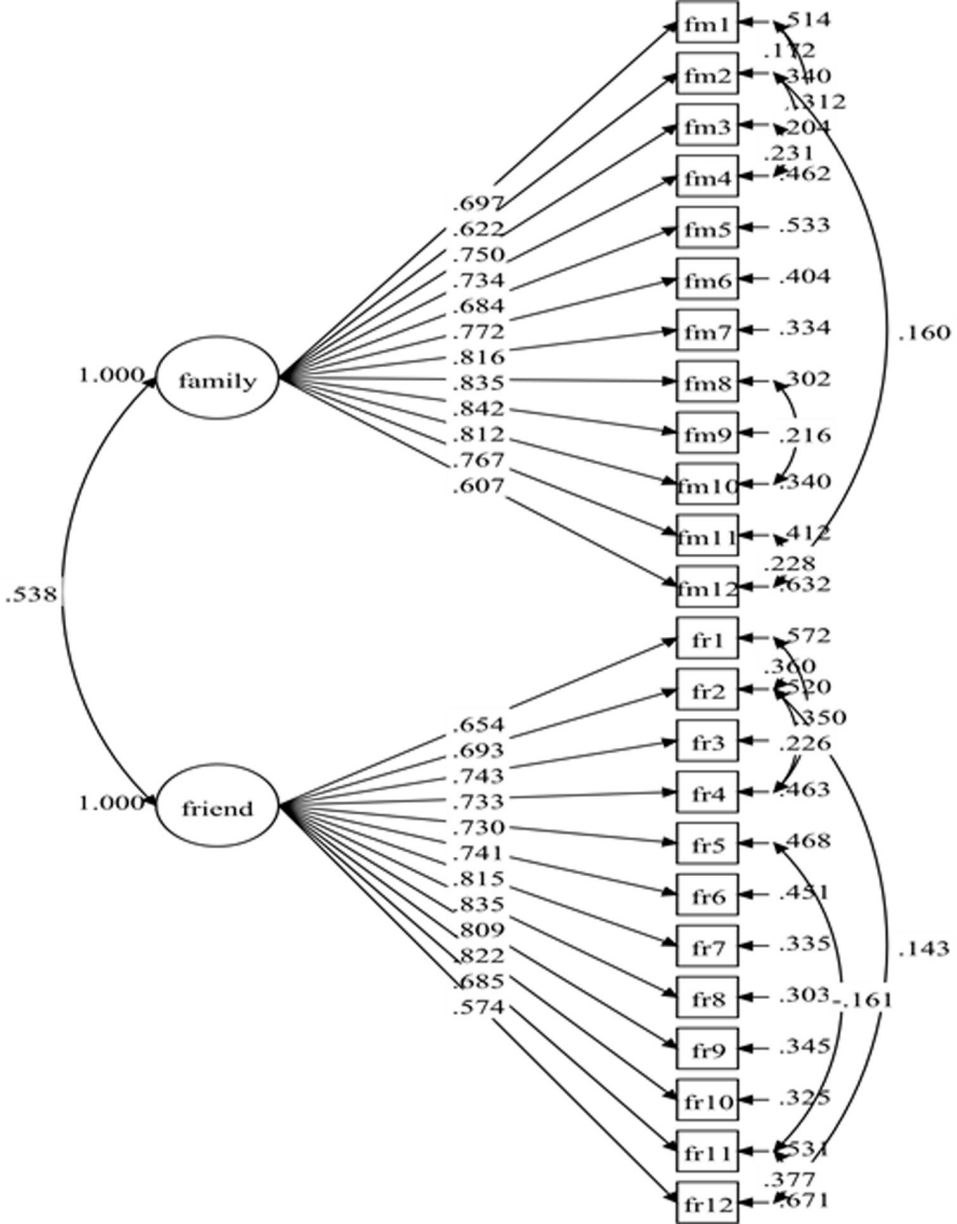
Social support measurement model (Model-2).

The initial specified measurement model for social support comprised of 24 items and two factors consisted with previous studies [11, 25, 26]: family support (12 items) and friend support (12 items). The results of the initial specified measurement model (Model-1) show poor fit indices (Table 1). Nonetheless, all the items have standardized factor loading of .50 and greater with *p*-value < 0.001 (Fig 1). The model fit indices were improved after adding 14 pairs of covariances between residuals' items within the same factor (Fig 2). The fit indices of the re-specified model (Model-2) were desirable (Table 1) with all the items retained. The result for Model-2 showed standardized item loading ranging from .574 to .842, which were considered as moderate to very good (Fig 2).

**Table 1:** Summary for social support model fit indices.

Path model	RMSEA (90% CI)	CFI	TLI	SRMR
Model-1	.075 (.072, .079)	.887	.876	.058
Model-2^a^	.061 (.057, .064)	.932	.920	.054

^a^Model-2 with correlated items residual; FR12 with FR11, FR2 with FR1, FM3 with FM1, FR4 with FR1, FM10 with FM8, FM4 with FM1, FR4 with FR2, FM12 with FM11, FM4 with FM3, FM4 with FM2, FM2 with FM1, FM12 with FM2, FR12 with FR2, FR11 with FR5.

### Physical environment for physical activity measurement model

**Fig 3:**
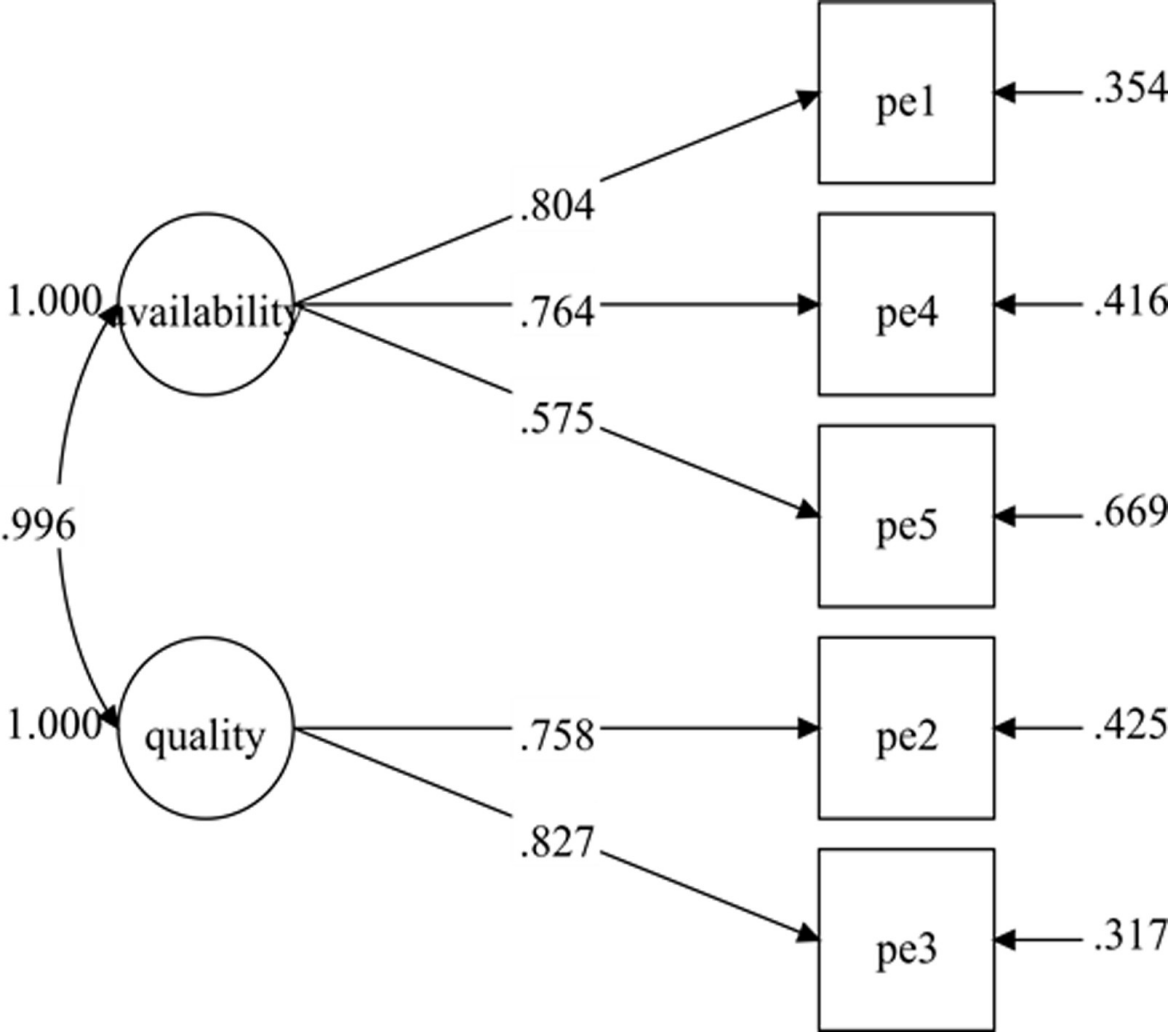
Physical environment measurement model (Model-1).

**Fig 4:**
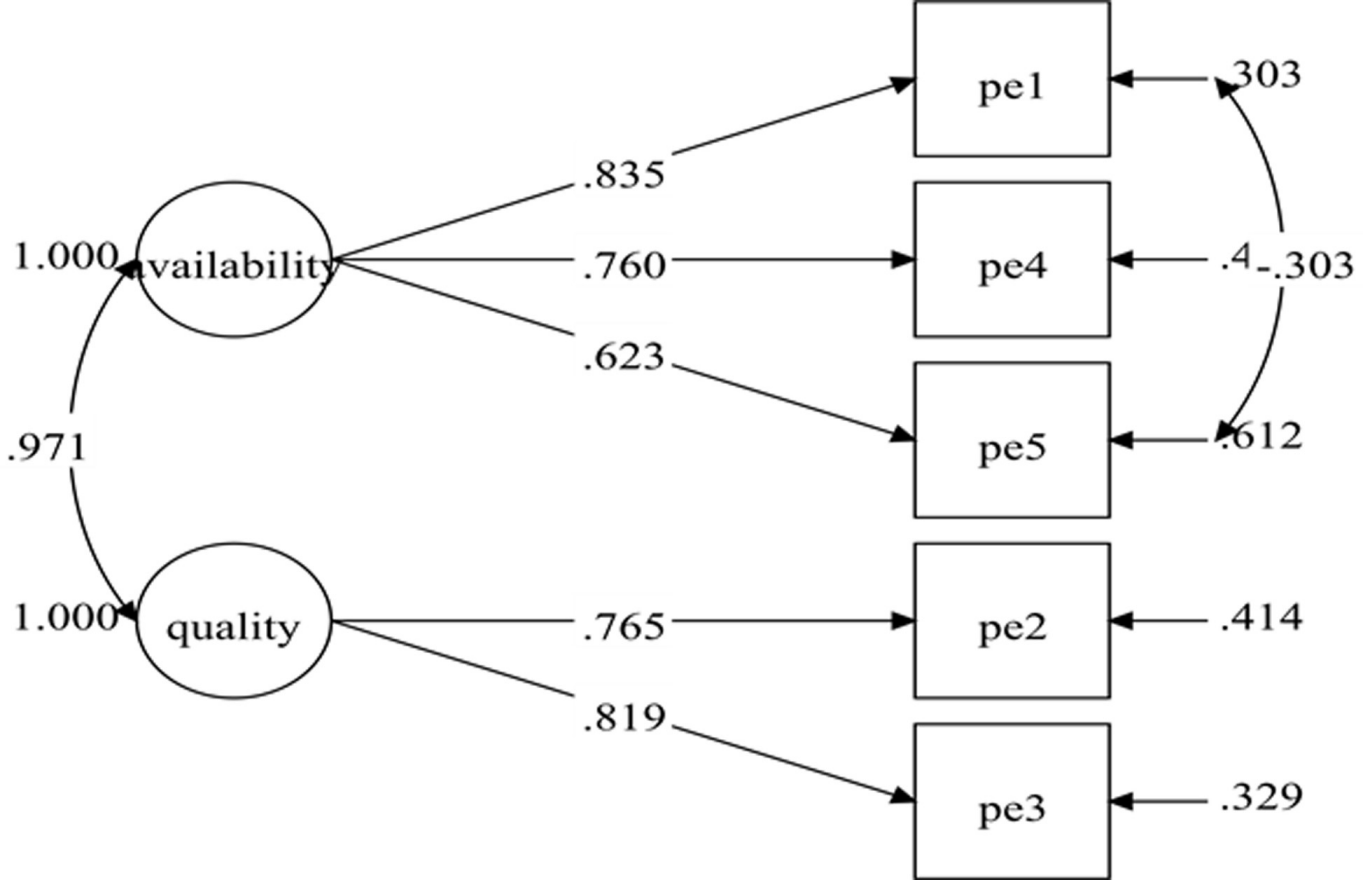
Physical environment measurement model (Model-2).

The initial specified measurement model for physical environment comprised of five items and two factors consistent with previous studies [11, 25, 26]: the availability of exercise facilities (three items) and the quality of exercise facilities (two items). The results of the initial specified measurement model (Model-1) show poor fit indices (Table 2). Nonetheless, all the items have standardized factor loading of .50 and greater with *p*-value < .001 (Fig 3). The model fit indices were improved after adding one pair of covariances between residuals' items within the same factor (Fig 4). The fit indices of the re-specified model (Model-2) were desirable (Table 2) with all the items retained. The result for Model-2 showed standardized item loading ranging from .623 to .835, which was considered as good to very good (Fig 4).

**Table 2:** Summary for physical environment model fit indices.

Path model	RMSEA (90% CI)	CFI	TLI	SRMR
Model-1	.098 (.071, .128)	.975	.937	.025
Model-2^a^	.054 (.021, .092)	.994	.981	.013

^a^Model-2 with correlated items residual; PE5 with PE1.

### Composite reliability (CR) and discriminant validity

Within social support, the CR was .918 for family support and .919 for friend support. The AVE was .560 for family support and .547 for friend support. The CR and AVE values were higher than the recommended values of .60 and .50 respectively, hence it was concluded that the social support scale for exercise showed sufficient convergent validity [37]. The squared correlation coefficient of family support and friend support was .054, which is less than the AVE values of .560 and .547. Hence, the social support scale has sufficient discriminant validity [37]. Table 3 presents the CR and AVE values, the correlation coefficients, and the square of the correlation coefficient of the final social support for the exercise model.

**Table 3:** Composite reliability (CR), average variance extraction (AVE), factor correlation and squared correlation for social support final model.

Variables	CR (95% CI)	AVE	1	2	*r*^*2*^
1. Family support	.918 (.908, .928)	.560	1	.233**	.054
2. Friend support	.919 (.909, .929)	.547		1	

**Correlation is significant at the .001 level (two tailed), *r*^*2*^ = squared correlation coefficient.

For the physical environment, the CR was .813 for perceived availability of exercise facilities and .771 for perceived quality of exercise facilities. The AVE was .554 and .628 for perceived availability and perceived quality, respectively. The CR and AVE values were higher than the recommended values of .60 and .50 respectively, thus it was concluded that the physical environment scale for physical activity showed sufficient convergent validity [37]. The squared correlation coefficient of perceived availability and perceived quality was .411, which is less than the AVE values of .554 and .628. Hence, the physical environment scale has sufficient discriminant validity [37]. Table 4 presents the CR and AVE values, the correlation coefficients, and the square of the correlation coefficient of the final physical environment for the physical activity model.

**Table 4:** Composite reliability (CR), average variance extraction (AVE), factor correlation and squared correlation for physical environment final model.

Variables	CR (95% CI)	AVE	1	2	*r*^*2*^
1. Availability	.813 (.779, .847)	.554	1	.641**	.411
2. Quality	.771 (.733, .810)	.628		1	

**Correlation is significant at the .001 level (two tailed), *r*^*2*^ = squared correlation coefficient.

### Test-retest reliability

A total of 120 participants completed both the social support scale and the physical environment scale twice within the interval of 14 days. Their mean score for social support increased from 74.3 (SD = 17.3) at day one to 76.0 (SD = 17.4) at day 14. The ICC was .920 for family support (95% CI, .887, .943, *p*-value < .001), and .984 for friend support (95% CI, .977, .989, *p*-value < .001). For the physical environment, their mean score increased from 17.2 (SD = 3.6) at day one to 17.8 (SD = 3.7) at day 14. The ICC was .895 for perceived availability (95% CI, .853, .926, *p*-value < .001), and .774 for perceived quality (95% CI, .691, .837, *p*-value < .001). These results showed adequate stability [40].

### Internal consistency

For the social support scale, the Cronbach’s alpha was .940 for family support and .936 for friend support. For physical environment scale, the Cronbach’s alpha obtained was .743 for perceived availability and .771 for perceived quality. Although there is no precise standard for the level of correlation, a recommended guideline is that items that correlate below .3 with the total score are considered less adequately related and consequently contribute less to the measurement of the factor [41]. The item-correlation values for all of the items ranged between .582 to .797 and between .470 to .654 for social support and physical environment scales respectively.

## Discussion

In this study, the researchers translated the English versions of the social support and physical environment scales into Malay and then ascertained the validity and reliability of the Malay versions of these scales among undergraduate students in USM using confirmatory factor analysis. The results obtained showed acceptable evidence of the validity and reliability of the social support scale for exercise and physical environment scale for physical activity scales within the data studied. The scales fit the data well and provided solid evidence for construct validity.

The social support and physical environment models verified in the present study were shown to have adequate internal reliability with the study sample. The Cronbach's alpha obtained for social support (.940 for family support and .936 for friend support) and for the physical environment (.743 for perceived availability and .771 for perceived quality) appeared to be consistent with the previous study (.85 for family support and .88 for friend support) and .78 for the physical environment [11]. All item-total correlation values were higher than .30, which indicate that the scales have sufficient internal reliability, and each item contributed to the measurement of its core factor [41]. The ICC values for test-retest obtained in this study were all above .70. As such, all scales have sufficient stability [40]. Yang et al., [25] reported test-retest values of .83 for family support; .89 for friend support; and .91 for physical environment [11].

CFA was conducted to investigate and confirm the factor models of social support and physical environment by assessing the measurement model validity. The social support scale was specified with 24 items and two factors, and the physical environment scale was specified with five items and two factors. The CFA results confirmed that both models had a good fit with the data obtained from Malaysian undergraduate students, as no items were deleted from the original version. The convergent validity of the models assessed using CR and AVE were both above the recommended values of .60 [36] and .50 [37] respectively. These suggest that both models have adequate convergent validity and or stable precision of estimation [37, 42]. In addition, all correlations between the factors in the two models were less than the cut-off value of .85. The squared correlation coefficients were all less than the AVE values of its factors. These results showed that the factors in the social support model and the physical environment model do not overlap much and each factor explains a distinct variance than the other factor [37].

The previous studies [23, 24, 25] on the social support scale for exercise and the physical environment scale for physical activity scales used in this study did not state whether covariances between error residuals were added or not. However, in the present study 14 pairs of covariances between residuals' items (six for friend support and eight for family support) were added for social support scale and one pair was added for the physical environment scale (i.e., perceived availability). These residuals covariances were added within the same factor for each scale based on the MI values reported in Mplus output and after adequate theoretical support was carried out by the researchers. Among the 14 pairs of residual covariances added for the social support, five similar pairs were added to both the family support and friend support including (1) FM1/FR1 (“Exercised with me”) with FM2/FR2 (“Gave me encouragement to stick with my exercise program”), (2) FM1/FR1 (“Exercised with me)” with FM4/FR4 (“Tries to exercise with me”), (3) FM2/FR2 (“Gave me encouragement to stick with my exercise program”) with FM4/FR4 (“Tries to exercise with me”), (4) FM2/FR2 (“Gave me encouragement to stick with my exercise program”) with FM12/FR12 (“Praise me about the changes in my body that I got from exercising”), and (5) FM11/FR11 (“Doing simple tasks for me to have more time for exercise”) with FM12/FR12 (“Praise me about the changes in my body that I got from exercising”). For the physical environment scale, a negative covariance between residual for PE1 (“There are enough exercise facilities and places such as jogging track, cycling path, parks, playgrounds parks, or gyms”) with PE5 (“The district office provides various physical activity programs like exercising class or sport events for the residents”) was added for the perceived availability factor. According to Kline. [38], residual covariances in CFA represent the assumption that the two paired items share some unexplained variances in common that is not defined in the model. Positive values of correlated residual terms indicate that the initial model underestimates the specific items covariance, whereas negative values indicate that the model overestimates this covariance [43]. The negative residual covariance between PE1 and PE5 could occur since both items were asking about the availability of exercise facilities and PE1 did not specify as to who provided the facilities, which could lead to most of the participants to perceive these items as similar. Additionally, in social psychological research, residual covariances should be included in the model when they make a substantive meaning [44, 45].

According to the social-ecological framework, social support (e.g. support from family and friends) and environmental factors (e.g. access and quality recreational facilities) have a major influence on children’s physical activity [46]. Some studies have shown that Malaysians values and cultures assign importance to family bonds, and that adolescents with adequate social support are more likely to succeed in life [47, 48]. The primary function of the Malaysian Ministry of Youth and Sports is the provision of training centers for youth registration of sports bodies, and physical environments (e.g., comfort level and temperature) was significantly related to civil servants productivity in Malaysia [49]. Given that, objective physical activity was found to be independently related to higher social support, lower age, more average hours of sunshine, and greater perceived behavioral control [50]. Hence, in this study, we presented standardized measures consistent with previous studies [23, 25, 26] that could provide a general understanding of social-environmental factors that may modify and enhance physical activity behavior among university students.

There are some limitations related to this study. Firstly, the survey was conducted at a single university, hence the results should be generalized with caution. However, the large sample size used may add more strength to the conclusions and findings of this study. Secondly, the use of a self-reported paper-based survey could produce response bias and reduce the accuracy of the data obtained. To control this intricacy, all the participants were guaranteed of the confidentiality of the data and advised to sincerely response to all the items regarding their true perception and not discuss with their friends when filling out the questionnaires. Also, the researchers suggest that future study could consider a probability sampling method rather than the convenience sampling method used in the present study to enhance the generalizability of the findings.

This study has shown clearly that the Malay versions of the social support scale for exercise and the physical environment scale for physical activity are suitable and consistent with previous studies in assessing physical activity behavior among university students. These scales were established to have good validity and reliability with all the items retained. Nevertheless, it is imperative to further assessed the replicability of social support and physical environment models in varying populations with different age ranges, education levels, occupations, and health conditions, not only university students. Also, it will be essential to study these scales among various Malay-speaking population.

## Conclusion

The final results provide psychometric evidence for using a questionnaire to evaluate perceived social-environmental factors that are associated with physical activity participation among university students in Malaysia. The Malay version of the social support scale for exercise and physical environment scale for physical activity are considered valid and reliable to be used among Malaysian university students. All items were retained and confirmed to be fit for the sample data. The final results could be beneficial to exercise educators, professionals, and policy makers for assessment and implementation of necessary plans to support and create awareness of the importance of engaging in regular physical activities. Researchers can utilize these tools to examine and explore the relationships between these social-environmental variables and other psychological variables to explain exercise behavior among the Malay-speaking population.

## Supporting information

S1 Appendix The Malay version of the social support scale for exercise and the physical environment scale for physical activity. (PDF)

S1 Data (PDF)
