# Supplementary material for: The validity and reliability of the Malay version of the social support for exercise and physical environment for physical activity scales
Source: PLoS One. 2020 Sep 28;15(9):e0239725. doi: 10.1371/journal.pone.0239725 (PMC7521693; doi:10.1371/journal.pone.0239725)
Supplement: S1 Appendix — (PDF) [file pone.0239725.s001.pdf]

Appendix:

The Malay Version of the Social Support Scale for Exercise and the Physical Environment Scale for Physical Activity

Skala Sosial Sokongan

Arahan: *Sila berikan penilaian dengan skala 1,2,3,4 atau 5 bagi pernyataan berikut mengikut kesesuaian anda.*

	**Keluarga saya...**	Tiada (1)	Jarang (2)	Beberapa Kali (3)	Selalu (4)	Sentiasa (5)
1.	Bersenam dengan saya.	1	2	3	4	5
2.	Memberi saya sokongan untuk sentiasa kekal dengan program senaman saya.	1	2	3	4	5
3.	Mengubah jadual mereka supaya dapat melakukan senaman bersama.	1	2	3	4	5
4.	Cuba untuk bersenam dengan saya.	1	2	3	4	5
5.	Membantu mengingatkan untuk melakukan senaman (“Adakah anda pergi bersenam malam ni?”).	1	2	3	4	5
6.	Merancang untuk melakukan rekreasi senaman luaran.	1	2	3	4	5
7.	Berbincang senaman dengan saya.	1	2	3	4	5
8.	Berbincang tentang betapa mereka suka melakukan senaman bersama.	1	2	3	4	5
9.	Membantu merancang aktiviti berdekatan dengan senaman saya.	1	2	3	4	5
10.	Berbincang tentang betapa mereka suka melakukan senaman.	1	2	3	4	5
11.	Melakukan tugasan mudah untuk saya supaya saya mempunyai lebih masa untuk bersenam.	1	2	3	4	5
12.	Memuji saya tentang perubahan bentuk badan saya hasil daripada melakukan senaman.	1	2	3	4	5

	Kawan saya...	Tiada (1)	Jarang (2)	Beberapa Kali (3)	Selalu (4)	Sentiasa (5)
1.	Bersenam dengan saya.	1	2	3	4	5
2.	Memberi saya sokongan untuk sentiasa kekal dengan program senaman saya.	1	2	3	4	5
3.	Mengubah jadual mereka supaya dapat melakukan senaman bersama.	1	2	3	4	5
4.	Cuba untuk bersenam dengan saya.	1	2	3	4	5
5.	Membantu mengingatkan untuk melakukan senaman (“Adakah anda pergi bersenam malam ni?”).	1	2	3	4	5
6.	Merancang untuk melakukan rekreasi senaman luaran.	1	2	3	4	5
7.	Berbincang senaman dengan saya.	1	2	3	4	5
8.	Berbincang tentang betapa mereka suka melakukan senaman bersama.	1	2	3	4	5
9.	Membantu merancang aktiviti berdekatan dengan senaman saya.	1	2	3	4	5
10.	Berbincang tentang betapa mereka suka melakukan senaman.	1	2	3	4	5
11.	Melakukan tugasan mudah untuk saya supaya saya mempunyai lebih masa untuk bersenam.	1	2	3	4	5
12.	Memuji saya tentang perubahan bentuk badan saya hasil daripada melakukan senaman.	1	2	3	4	5

Skala Persekitaran Fizikal

Arahan: *Sila berikan penilaian dengan skala 1,2,3,4 atau 5 bagi pernyataan berikut mengikut kesesuaian anda.*

	**Dalam komuniti saya...**	Sangat tidak setuju (1)	Agak tidak setuju (2)	Neutral (3)	Agak setuju (4)	Sangat setuju (5)
1.	Terdapat kemudahan tempat dan fasiliti senaman yang mencukupi. (seperti; trek joging, laluan berbasikal, taman-taman, taman permainan, atau gim).	1	2	3	4	5
2.	Kemudahan fasiliti dan tempat adalah terjaga rapi dan selamat untuk digunakan pada waktu malam.	1	2	3	4	5
3.	Ia menyediakan peluang yang mencukupi untuk menggunakan fasiliti senaman dengan harga yang murah atau secara percuma.	1	2	3	4	5
4.	Kemudahan tempat dan fasiliti senaman adalah berdekatan.	1	2	3	4	5
5.	Pejabat daerah menyediakan pelbagai program aktiviti fizikal seperti kelas senaman atau acara sukan untuk penduduk.	1	2	3	4	5
