# Supplementary material for: The validity and reliability of the Malay version of the social support for exercise and physical environment for physical activity scales
Source: PLoS One. 2020 Sep 28;15(9):e0239725. doi: 10.1371/journal.pone.0239725 (PMC7521693; doi:10.1371/journal.pone.0239725)
Supplement: S1 Data — (PDF) [file pone.0239725.s002.pdf]

ID	Gender	Ethnic	Age	Sport frec	Sport dur	SE FM1	SE FM2	SE FM3	SE FM4	SE FM5	SE FM6	SE FM7	SE FM8	SE FM9	SE FM10	SE FM11	SE FM12	SE FR1	SE FR2	SE FR3	SE FR4	SE FR5	SE FR6	SE FR7	SE FR8	SE FR9	SE FR10	SE FR11	SE FR12	PE1	PE2	PE3	PE4	PE5
1	1	2	20	2	120	3	3	3	4	2	3	3	2	2	3	3	4	3	2	3	3	3	3	3	4	3	4	3	4	4	4	2	2	3
2	1	2	20	2	60	4	5	4	3	2	2	3	5	4	4	2	5	2	2	4	3	2	2	3	3	5	4	2	2	3	2	5	3	2
3	1	2	21	4	120	3	2	4	5	3	3	2	2	2	1	3	3	3	3	4	3	3	3	2	2	3	3	3	3	4	3	2	4	3
4	2	1	19	1	60	3	4	3	4	4	4	2	3	3	3	3	4	4	4	3	4	3	4	3	4	4	3	4	4	3	3	3	3	3
5	1	1	19	7	120	2	4	2	3	4	3	4	4	3	3	4	3	4	4	4	4	4	4	4	4	4	4	4	4	3	2	3	2	2
6	1	1	19	4	40	2	4	3	3	4	3	4	3	3	3	3	4	5	4	4	5	5	5	4	5	4	5	4	4	5	5	5	5	4
7	2	1	19	2	45	2	3	1	3	1	1	1	3	3	3	3	3	3	3	3	3	3	3	3	3	2	3	2	2	4	4	4	4	4
8	2	3	26	1	30	1	3	2	1	1	1	3	3	1	1	2	3	2	4	2	3	3	5	3	5	5	5	2	1	1	1	1	1	2
9	2	1	19	1	30	2	4	4	3	4	3	2	2	2	2	2	4	4	3	4	3	4	4	3	4	3	4	4	3	3	4	4	3	4
10	1	3	19	3	23	2	3	2	3	4	3	3	3	3	3	3	3	4	4	4	4	4	4	3	3	3	4	4	3	3	3	4	4	3
11	2	1	22	1	20	2	2	3	3	1	3	3	2	2	1	1	3	2	1	1	3	1	2	2	1	2	1	1	2	5	3	4	4	3
12	2	1	21	1	30	2	4	3	5	3	3	2	3	3	3	3	3	4	5	5	5	5	5	5	5	5	5	5	5	4	2	5	5	2
13	2	1	22	1	30	2	3	2	3	2	4	4	3	2	3	3	3	2	3	2	3	4	4	4	4	4	4	4	3	2	3	2	3	4
14	2	1	21	1	30	3	4	3	4	4	4	5	4	5	4	4	4	3	5	3	4	5	3	4	4	3	4	3	5	5	3	5	5	5
15	1	2	20	7	60	2	3	2	2	2	2	2	2	2	2	2	2	3	2	2	2	3	2	2	3	3	3	2	3	4	3	4	4	2
16	1	2	21	3	90	1	2	1	1	1	1	2	1	2	1	3	3	2	3	1	1	2	2	3	2	2	3	3	2	4	4	5	4	3
17	1	2	21	1	120	2	3	3	3	4	2	2	3	3	3	3	2	3	3	3	3	3	3	3	3	3	3	3	3	3	3	3	3	3
18	2	4	19	2	180	2	5	2	4	4	3	3	1	3	1	1	3	4	4	4	4	4	4	4	4	4	4	1	1	1	1	2	1	4
19	1	2	21	3	60	2	2	3	4	4	2	2	3	3	3	5	5	4	4	5	5	3	5	5	4	1	1	3	3	3	2	2	1	1
20	1	2	21	1	30	1	4	1	1	2	1	1	1	1	1	1	3	3	4	4	5	1	1	1	2	2	2	3	2	4	4	4	4	4
21	1	2	21	3	60	3	4	3	4	2	3	4	5	3	4	3	4	3	4	2	3	4	5	4	4	3	3	4	5	4	3	3	2	4
22	2	2	19	2	120	2	3	4	4	2	4	4	3	5	5	4	3	3	4	3	4	3	5	5	5	4	5	3	4	3	3	5	2	3
23	1	2	20	2	120	2	3	3	2	4	3	3	4	3	3	3	3	4	4	3	3	4	3	3	3	4	3	2	3	3	3	2	3	3
24	1	2	23	7	45	4	5	3	5	3	4	3	5	5	3	4	4	3	4	3	4	4	5	5	3	4	4	3	4	5	4	3	2	3
25	2	3	20	1	120	2	3	2	3	2	2	4	5	3	4	4	2	2	3	3	2	4	3	3	4	2	3	2	3	3	2	3	4	4
26	2	2	22	7	90	4	5	3	5	2	4	3	2	4	3	3	3	2	3	2	4	3	4	4	3	4	3	5	4	5	5	4	5	4
27	2	2	20	1	60	2	3	2	3	2	2	2	2	2	2	3	3	3	4	3	3	2	4	3	3	3	3	3	3	3	3	4	4	3
28	1	2	19	2	60	3	4	3	4	4	3	4	5	4	3	5	4	4	5	4	3	4	3	5	4	4	3	3	5	3	4	3	3	4
29	2	2	20	1	120	3	4	3	2	3	3	2	4	4	5	3	2	3	4	2	2	3	5	2	1	3	3	4	2	3	4	5	5	4
30	2	4	19	2	90	1	1	1	1	1	1	1	1	1	1	1	1	2	1	1	1	1	1	1	1	1	1	1	1	3	3	3	3	3
31	2	3	19	2	60	3	4	3	4	4	4	4	4	4	3	4	3	4	4	4	4	4	3	4	4	4	4	4	4	4	4	4	4	4
32	2	1	23	1	30	3	5	3	5	4	5	4	5	5	4	4	3	3	4	3	3	5	4	3	4	4	5	3	3	4	4	5	5	5
33	2	1	19	2	30	2	3	2	2	2	3	2	3	2	3	3	3	2	2	2	2	2	2	2	2	2	2	2	2	3	4	4	3	3
34	1	2	20	12	30	2	3	1	2	3	2	2	2	1	2	2	3	4	3	2	3	3	2	3	3	2	2	1	3	5	4	4	3	2
35	1	2	24	1	60	2	3	2	1	2	1	2	3	2	1	1	2	4	5	4	5	3	3	4	4	4	4	3	3	3	4	4	4	2
36	2	2	19	2	60	3	5	3	3	5	2	3	3	4	3	3	5	5	5	4	4	4	3	4	5	3	3	4	3	3	3	2	3	2
37	1	3	20	2	60	4	3	4	5	5	4	4	3	3	4	4	5	5	2	2	3	4	4	5	5	5	5	4	1	3	4	4	5	1
38	1	1	20	1	60	4	4	4	4	4	4	4	4	4	4	4	4	4	3	4	3	4	4	4	3	3	4	3	4	3	3	3	3	3
39	1	1	20	4	260	2	4	4	4	4	4	4	4	4	4	4	4	4	4	4	4	4	4	4	4	4	4	4	4	3	3	3	3	3
40	1	1	20	3	20	1	2	1	1	1	1	1	1	1	1	3	3	3	3	2	3	2	3	3	3	2	4	2	4	3	2	2	1	1
41	1	1	21	4	60	2	4	2	4	3	4	2	2	3	2	2	3	3	3	2	3	2	3	3	3	3	3	2	2	3	3	3	3	2
42	1	1	20	1	120	4	4	5	5	5	5	5	5	5	5	5	5	1	1	1	1	1	1	1	1	2	2	2	2	5	5	5	5	5
43	2	3	19	2	30	3	5	3	5	3	4	4	4	4	5	5	4	3	4	4	4	4	4	4	4	4	4	4	4	5	4	5	5	4
44	2	3	20	1	120	4	3	3	3	4	2	3	4	2	2	2	2	4	4	3	3	3	3	3	3	3	4	4	4	4	4	4	3	3
45	1	3	21	3	120	3	3	3	3	4	3	4	4	3	4	3	4	3	3	3	4	3	2	3	4	3	3	4	3	3	3	4	3	3
46	1	3	22	3	120	4	3	3	3	4	3	4	3	4	3	3	4	4	4	3	3	3	3	4	3	3	4	3	3	3	4	3	4	3
47	2	2	21	1	30	3	3	3	3	3	3	3	2	2	2	2	3	3	3	2	3	2	2	2	2	1	2	1	3	3	2	2	3	2
48	1	1	19	4	60	2	2	3	4	2	3	4	1	2	2	2	2	2	1	2	3	2	2	1	3	2	1	3	2	3	4	3	4	3
49	2	2	20	1	30	3	4	2	2	4	3	2	3	3	3	4	4	4	4	4	5	4	4	3	5	4	4	3	5	4	3	3	3	2
50	2	1	22	1	60	3	4	2	3	3	3	3	3	3	3	2	2	3	2	3	3	3	2	3	3	1	2	3	2	3	3	4	2	1
51	1	2	22	3	45	3	5	2	3	4	2	3	3	3	3	1	4	4	4	2	3	3	3	3	3	3	3	2	4	3	3	3	3	1
52	1	2	20	2	45	2	5	2	3	3	3	2	2	2	2	3	4	4	4	4	4	4	1	4	4	4	4	1	4	3	3	3	3	1
53	1	2	19	2	60	1	4	1	1	3	2	1	1	1	1	2	4	3	3	2	3	3	1	3	3	2	3	2	3	4	4	4	4	2
54	2	2	19	2	60	1	1	1	2	2	3	3	2	3	1	2	1	2	2	1	2	3	3	3	1	2	2	2	2	3	2	1	1	3
55	1	1	21	3	45	1	3	1	2	2	2	2	2	1	2	1	3	2	3	1	2	2	1	1	3	3	3	2	4	4	3	4	3	2
56	1	1	20	1	50	1	3	1	1	3	1	1	1	2	1	2	4	2	3	2	2	2	1	2	2	2	2	1	3	2	2	2	2	1
57	2	1	19	1	60	3	3	2	2	3	3	3	2	2	2	2	5	3	4	3	4	3	2	3	3	2	3	2	4	2	2	2	2	1
58	1	2	21	3	30	3	2	2	3	2	1	1	1	1	1	1	3	2	2	2	2	1	2	3	3	2	1	1	3	4	2	4	4	3
59	2	1	20	2	60	4	4	3	4	3	4	4	4	4	3	3	4	3	4	2	3	3	4	5	4	4	4	3	4	5	5	5	5	3
60	2	2	22	1	20	2	4	2	3	3	2	2	2	2	3	2	2	3	2	3	3	2	3	2	3	3	3	3	2	4	3	4	4	2
61	1	1	20	5	60	2	1	2	2	1	2	2	3	2	2	1	2	3	4	3	4	4	3	3	3	3	2	4	3	5	3	5	3	3
62	1	1	22	2	20	3	3	3	2	2	4	1	2	4	2	3	3	3	2	2	3	4	3	3	3	2	3	4	3	3	4	3	2	3
63	1	1	21	7	60	1	2	1	2	3	2	1	1	1	1	1	1	4	4	4	4	5	4	4	4	4	4	1	2	3	4	2	4	3
64	2	1	20	1	120	4	5	4	4	4	4	3	4	3	3	3	5	5	5	3	4	4	4	4	3	4	3	4	4	4	4	4	4	4
65	2	1	20	1	30	3	4	3	3	4	4	3	3	3	4	4	4	3	4	2	4	4	4	4	3	3	4	3	2	4	3	4	4	4
66	1	1	20	4	90	3	4	3	4	3	4	4	3	4	4	3	4	3	4	3	3	3	4	4	4	4	4	4	4	3	4	4	3	4
67	1	1	20	5	30	2	3	2	3	4	2	3	1	1	1	2	2	3	4	2	4	4	3	4	4	3	3	2	4	3	3	2	2	1
68	2	2	20	1	20	3	2	2	2	2	2	2	2	3	2	3	3	3	3	2	2	1	2	2	2	2	2	2	2	3	2	2	1	1
69	1	2	20	14	10	3	2	4	3	3	3	3	3	3	4	3	2	2	2	3	3	2	3	3	2	2	2	1	1	3	3	3	1	1
70	1	2	20	2	180	3	3	4	4	3	4	3	3	3	4	4	4	4	4	4	4	4	3	3	3	2	2	1	1	3	3	4	4	1
71	1	2	20	2	30	3	3	3	3	4	4	3	4	3	3	4	3	2	2	3	3	3	3	2	3	3	4	3	3	2	2	2	3	3
72	1	2	20	1	15	3	4	3	3	2	3	2	3	2	1	1	1	3	2	2	3	3	3	2	2	3	3	2	2	1	3	3	3	3
73	2	2	20	1	60	1	3	1	3	3	2	4	2	1	4	4	1	4	4	3	4	3	2	4	4	2	4	2	1	3	2	3	3	1
74	1	2	20	4	45	3	4	1	2	3	4	3	2	3	4	3	5	5	5	5	5	4	4	5	5	4	5	3	5	5	5	5	5	3
75	2	3	19	2	20	3	4	3	3	2	4	3	2	3	2	4	4	3	3	2	3	2	3	3	3	3	3	2	3	4	3	4	4	4
76	1	2	18	5	120	1	1	1	1	1	1	1	1	1	2	2	1	5	4	4	4	4	2	3	3	5	3	3	3	2	3	4	2	2
77	2	3	19	2	60	3	5	4	3	4	3	5	5	5	4	4	4	2	4	4	4	3	4	5	5	3	4	4	5	5	5	4	5	2
78	2	1	20	2	45	4	4	1	5	4	3	5	5	3	3	4	5	2	3	1	2	5	4	4	3	4	4	2	4	5	3	2	4	4
79	2	1	19	2	40	4	3	4	4	4	3	3	3	4	4	3	4	4	4	4	3	4	5	4	4	4	3	4	4	4	3	3	3	4
80	2	3	19	2	40	4	4	2	4	3	4	4	4	4	4	4	4	4	4	2	4	3	4	4	4	3	3	4	3	4	4	3	4	4
81	2	1	20	2	105	2	3	1	3	1	1	1	1	1	2	2	1	3	4	1	2	2	2	2	1	2	1	1	1	3	3	3	3	3
82	2	1	20	2	105	4	2	2	4	4	2	2	3	2	2	2	1	3	4	4	4	4	4	4	4	3	4	3	2	4	2	4	3	3
83	2	1	19	2	90	2	5	4	4	4	4	4	4	3	3	4	2	5	5	5	5	5	5	5	5	5	5	5	3	5	4	4	5	2
84	2	1	19	2	90	2	4	1	2	1	1	1	2	2	2	3	1	4	3	4	4	3	3	2	3	3	4	1	1	3	2	3	3	3
85	2	1	20	2	20	3	4	2	3	4	5	4	3	2	3	3	4	3	3	2	3	5	4	3	2	3	2	3	4	4	2	4	5	3
86	2	1	20	2	90	3	4	3	3	4	4	3	3	3	3	3	4	4	4	4	4	4	4	4	4	4	4	4	4	3	2	4	4	4
87	2	1	20	2	120	3	3	3	4	4	3	2	3	3	4	3	3	2	2	2	2	3	2	2	2	2	2	2	2	3	2	2	3	3
88	1	1	21	5	120	3	4	3	4	4	3	3	3	3	4	4	5	1	4	3	3	3	4	4	3	3	4	4	5	3	2	4	4	4
89	2	1	20	1	30	2	4	3	3	2	3	4	3	2	3	2	4	4	4	3	4	4	4	4	3	3	3	2	4	2	2	3	2	2
90	2	1	20	1	60	2	3	4	4	3	2	2	2	3	3	4	5	2	4	4	4	3	3	3	2	2	4	4	4	5	5	5	5	5
91	1	1	21	3	60	3	3	3	3	2	3	3	4	3	4	3	3	4	3	3	4	2	3	3	3	3	3	3	3	3	3	3	3	3
92	2	2	20	1	120	3	4	3	3	4	5	4	4	5	3	3	5	2	3	4	3	3	5	4	4	4	4	3	2	5	5	5	5	4
93	1	1	24	5	30	3	4	3	4	5	5	4	3	5	4	5	3	4	5	5	5	4	5	5	4	4	5	4	5	4	3	4	4	4
94	1	1	22	2	30	3	4	3	3	4	5	3	3	4	4	5	5	3	3	4	5	4	4	4	4	4	4	4	4	3	4	4	3	4
95	1	3	21	5	90	4	4	5	5	5	4	4	5	5	5	5	5	5	5	4	4	5	5	5	4	4	4	4	5	5	5	5	5	5
96	1	2	23	3	15	3	4	3	2	1	3	3	2	2	3	3	3	1	1	1	1	1	1	2	2	2	2	3	3	5	5	5	5	5
97	2	1	20	2	90	4	3	4	4	3	3	4	3	4	4	4	4	2	2	2	3	2	3	3	3	4	4	4	3	5	5	5	5	4
98	2	1	19	2	30	4	3	4	3	2	4	3	2	4	3	3	4	3	4	3	2	2	2	3	2	3	2	3	2	3	4	3	4	1
99	2	1	19	2	60	2	3	2	4	2	3	2	1	1	1	1	2	4	2	1	2	3	2	1	1	2	3	2	3	4	4	3	4	1
100	1	1	21	1	60	4	3	1	4	2	4	3	2	3	1	4	2	5	4	2	4	2	4	3	3	1	2	4	1	5	4	5	5	5
101	1	1	22	5	60	2	3	2	1	2	2	2	1	2	2	1	2	4	3	4	3	4	3	4	2	3	4	3	4	4	3	2	4	1
102	1	1	19	2	60	2	3	4	3	3	3	3	4	3	2	3	2	2	3	3	3	4	3	4	4	3	3	3	3	3	4	3	2	1
103	2	1	19	2	60	3	4	3	3	2	3	3	2	3	3	4	3	3	4	4	3	4	3	4	3	4	3	4	3	4	4	3	2	1
104	1	1	19	2	120	3	4	1	3	3	3	3	2	3	3	4	3	3	4	4	4	3	4	3	4	2	4	4	3	3	2	3	4	2
105	1	1	21	4	120	3	4	2	2	3	2	3	2	2	3	2	2	4	3	4	4	3	3	4	3	4	3	2	3	3	4	3	2	1
106	2	1	18	1	30	3	3	3	3	3	3	3	3	3	2	2	2	3	3	3	3	3	3	3	3	2	2	3	3	3	3	3	3	3
107	2	1	19	1	60	3	4	4	4	4	3	3	3	3	4	3	4	3	3	3	3	3	3	3	3	3	3	3	3	4	4	4	4	4
108	2	3	19	2	120	3	4	3	3	3	4	4	4	5	3	4	4	5	5	4	4	5	4	4	3	4	5	4	5	5	5	4	5	4
109	2	1	19	1	180	4	4	2	3	4	4	3	3	4	2	2	3	4	4	3	4	3	4	4	3	4	3	2	2	3	3	4	4	3
110	2	1	19	2	90	4	4	4	3	2	4	3	4	3	3	3	4	4	4	3	4	4	4	3	3	3	4	2	3	5	3	3	4	3
111	2	3	19	2	90	3	5	3	3	3	2	3	3	3	3	3	3	4	4	3	4	4	4	4	4	4	4	3	4	4	4	4	4	3
112	2	1	20	2	60	3	3	2	2	2	3	3	2	2	3	2	3	4	4	3	3	3	4	2	3	3	3	3	3	3	3	3	4	4
113	2	1	22	1	35	3	5	4	3	3	4	3	3	3	3	3	2	2	1	2	2	1	2	1	1	1	1	1	1	3	2	3	4	1
114	2	1	18	2	90	3	4	2	4	4	4	2	3	4	3	3	3	4	3	3	3	3	4	3	4	3	3	2	2	5	4	4	4	3
115	2	1	19	2	30	3	5	2	3	2	3	2	2	3	3	4	5	5	5	2	4	2	3	2	3	3	3	3	5	4	3	3	2	2
116	2	1	19	2	30	4	4	4	4	4	4	4	4	4	4	4	4	4	4	4	4	4	4	4	4	4	4	4	4	4	3	4	3	3
117	2	1	19	2	30	4	4	4	4	4	4	4	4	4	4	4	4	4	4	4	4	4	4	4	4	4	4	4	4	4	3	4	3	3
118	2	1	19	1	20	1	3	2	2	1	2	2	1	1	2	1	1	3	2	1	2	2	2	3	3	2	2	1	1	3	2	2	3	2
119	2	1	19	2	30	2	2	2	2	2	2	2	2	2	2	2	2	2	1	1	2	1	1	2	1	2	2	1	2	2	2	3	3	2
120	2	1	20	1	30	3	3	1	3	2	3	3	3	2	1	2	1	3	3	2	3	2	2	1	1	3	3	3	3	3	3	2	3	1
121	1	1	22	2	60	2	2	2	2	2	3	3	2	2	2	2	2	3	2	2	2	2	2	2	2	2	3	3	3	4	4	4	4	3
122	1	1	20	4	60	3	4	3	3	5	4	4	5	3	3	3	4	4	4	3	5	4	4	4	4	4	4	3	4	5	4	4	4	4
123	1	1	21	5	60	2	3	3	3	2	2	3	3	3	2	4	3	2	3	3	3	3	4	3	2	3	4	3	3	3	2	3	4	3
124	2	1	20	1	45	2	3	3	3	1	3	3	3	2	2	2	1	4	3	4	4	4	4	4	4	2	3	2	1	2	1	1	1	1
125	1	1	20	7	120	3	5	3	5	4	5	5	5	5	5	4	4	5	5	5	5	5	4	5	5	5	5	5	5	5	5	5	5	5
126	2	1	19	2	30	4	3	4	4	3	4	4	5	5	3	4	5	3	3	4	3	4	5	5	5	5	4	4	3	5	5	5	5	5
127	2	1	20	2	30	3	3	3	4	4	3	3	4	3	3	4	4	3	3	3	3	4	4	4	3	3	4	3	4	3	3	3	3	3
128	2	1	19	2	180	3	5	3	3	4	3	3	3	3	3	5	4	5	5	5	5	5	5	5	5	5	5	5	5	4	3	4	5	4
129	2	1	19	1	30	2	4	2	3	2	3	3	3	3	3	3	4	4	4	4	4	4	4	4	4	4	4	4	5	4	4	4	4	4
130	2	1	20	1	30	5	5	5	5	5	5	5	5	5	5	5	5	4	4	4	4	4	4	5	5	5	4	4	5	5	5	5	5	5
131	2	1	19	2	90	4	4	4	4	4	4	4	4	4	4	4	4	5	4	4	4	4	4	4	4	4	4	4	4	5	4	5	4	4
132	1	1	20	2	120	2	4	2	2	4	4	4	2	2	2	2	3	4	4	3	4	5	4	4	4	4	4	3	2	3	3	1	2	2
133	1	1	18	3	45	2	4	2	3	3	3	2	2	2	2	3	1	4	4	3	4	4	3	3	3	3	4	4	2	4	3	3	3	3
134	1	1	23	4	60	2	3	3	3	2	2	3	3	2	2	2	3	4	4	4	4	4	3	3	4	3	2	4	3	3	3	3	3	2
135	1	1	21	5	70	3	3	3	2	2	2	3	3	2	4	2	3	4	4	4	4	4	4	4	4	4	4	4	4	3	3	3	3	4
136	1	4	21	4	60	4	3	3	3	3	3	4	3	4	4	3	3	4	4	4	4	4	4	4	4	4	4	4	4	3	3	4	3	3
137	1	1	19	3	60	3	3	2	3	2	3	3	3	2	4	2	2	4	3	3	3	2	3	3	3	3	3	2	4	3	3	3	3	3
138	2	1	20	2	30	1	1	1	1	1	1	1	1	1	1	1	1	2	1	1	1	1	1	1	1	1	1	1	1	4	1	4	4	1
139	2	1	23	1	30	3	2	3	2	2	2	3	2	2	2	2	3	3	3	2	3	2	2	2	3	2	2	2	3	2	1	2	2	2
140	1	1	20	2	30	2	2	2	2	2	2	2	2	2	2	2	2	3	4	3	3	3	2	3	3	2	3	3	3	2	2	2	2	2
141	2	1	20	1	120	3	5	3	3	4	3	3	3	4	3	3	4	4	5	4	4	5	4	4	4	4	4	3	3	4	2	2	3	3
142	1	2	24	2	120	3	4	2	3	5	3	2	3	2	2	3	4	4	5	4	3	4	4	5	3	4	4	5	5	3	4	3	1	3
143	1	1	22	5	90	3	3	3	4	3	4	4	3	3	3	3	3	4	4	3	4	3	4	4	4	4	4	4	3	4	5	5	4	3
144	1	1	22	2	120	3	3	3	3	3	3	3	3	2	2	3	3	4	4	4	4	4	4	4	4	4	4	3	3	4	4	4	4	2
145	2	1	21	1	22	2	4	2	2	1	3	3	3	2	2	1	1	5	4	3	5	4	4	3	3	3	4	2	3	5	4	5	4	3
146	1	1	23	2	90	2	3	2	2	2	3	3	3	2	2	3	3	4	4	4	4	3	3	4	4	4	4	3	3	4	4	4	4	2
147	1	1	22	2	90	2	3	2	2	2	3	3	3	2	2	3	3	4	4	4	3	3	3	3	4	4	3	3	3	4	4	4	4	2
148	1	1	22	4	90	3	3	2	2	2	3	3	2	2	2	2	3	4	4	4	4	4	3	4	4	3	4	3	3	4	4	4	4	2
149	2	1	21	1	150	4	4	4	3	3	4	4	4	4	4	4	4	4	4	4	4	4	4	4	4	4	4	4	4	3	3	4	4	4
150	2	1	21	1	90	2	4	3	3	2	3	3	3	3	2	2	2	4	4	2	4	2	3	4	3	3	3	3	3	4	3	3	4	3
151	2	1	21	1	120	3	5	5	5	4	4	5	5	5	4	4	5	4	4	3	4	4	4	5	5	4	4	5	5	3	4	4	3	1
152	2	1	20	1	75	4	4	4	4	4	4	4	4	4	4	4	4	4	4	4	4	4	4	4	4	4	4	4	4	5	5	5	5	5
153	2	1	20	2	30	3	3	3	4	3	4	4	4	4	4	4	4	4	3	3	4	3	4	2	4	2	2	2	4	4	4	4	4	4
154	1	1	18	4	60	4	5	4	4	4	3	3	3	3	3	3	4	4	4	4	4	4	4	4	4	4	4	4	4	2	2	1	1	1
155	2	1	20	1	5	3	4	3	3	4	4	4	2	3	2	2	2	4	3	2	3	2	3	3	3	3	3	3	3	5	5	5	5	5
156	2	1	18	2	45	3	4	2	4	4	4	3	4	2	3	3	3	2	3	2	4	4	4	2	2	2	3	2	3	5	5	5	4	4
157	2	1	19	2	90	3	4	1	4	1	3	2	2	2	3	1	1	2	3	1	3	3	3	1	1	1	1	1	1	5	3	4	5	3
158	2	1	22	1	30	3	5	2	3	3	2	2	2	2	3	2	2	3	4	4	4	4	4	4	4	4	4	4	4	1	1	1	1	2
159	2	1	22	1	#NULL!	2	2	3	3	5	2	2	2	2	2	3	5	5	5	3	5	5	4	5	5	5	4	4	5	3	3	4	2	2
160	2	1	21	1	30	2	3	2	2	2	2	2	3	2	2	3	2	1	1	1	2	2	2	1	1	2	1	1	1	3	3	3	2	3
161	2	1	23	1	30	3	3	2	2	2	2	3	2	3	2	3	3	3	3	2	3	2	3	3	2	2	2	2	2	2	1	2	2	2
162	2	1	20	2	20	4	4	3	4	4	5	4	5	5	5	5	5	5	5	4	4	5	4	5	5	5	5	5	5	5	5	4	5	4
163	2	1	20	2	120	3	4	3	4	2	4	2	3	4	4	3	5	5	4	3	4	3	3	3	4	4	5	5	5	5	5	4	5	3
164	2	1	23	1	60	3	3	2	3	2	3	2	2	2	3	2	2	3	3	2	3	3	3	3	3	2	3	2	2	4	4	4	3	4
165	2	1	20	2	30	3	4	3	4	5	3	3	3	3	3	2	2	3	3	3	3	3	3	3	3	3	3	3	3	4	3	3	4	2
166	2	1	19	2	30	1	2	1	1	1	2	1	1	1	1	1	1	3	2	2	2	2	1	2	2	2	2	2	1	2	2	1	3	3

167	2	4	19	2	105	1	1	1	1	1	1	1	1	1	1	1	1	2	2	2	2	2	2	2	2	2	2	2	2	3	3	3	3	3
168	2	2	19	1	30	3	4	4	5	3	5	5	3	5	3	3	3	3	4	4	4	3	4	4	3	3	4	3	3	3	2	3	4	3
169	2	1	20	1	30	3	3	3	3	3	3	3	3	3	3	3	3	3	3	3	3	3	3	3	3	3	1	3	2	4	4	4	4	4
170	1	1	19	3	40	2	4	2	2	3	2	2	2	2	3	2	3	4	4	4	4	4	4	4	4	4	4	4	4	5	3	3	4	3
171	1	2	20	3	120	2	3	2	2	2	2	2	2	2	2	3	2	3	3	2	3	4	3	2	3	3	2	3	4	2	3	2	2	3
172	2	1	19	1	30	3	4	2	3	2	3	2	3	2	3	3	3	4	4	2	4	3	3	3	3	3	3	4	4	4	4	5	4	4
173	2	3	19	1	60	3	4	4	4	4	3	3	4	3	4	3	4	4	4	4	4	4	4	4	4	4	4	4	4	5	5	5	5	5
174	2	1	19	1	60	3	4	4	4	4	3	3	4	3	4	3	4	4	4	4	4	4	4	4	4	4	4	4	4	5	5	5	5	5
175	2	1	19	2	60	2	3	2	2	2	3	2	2	2	2	2	3	2	3	3	3	3	4	2	4	3	3	3	4	4	1	2	2	2
176	2	1	18	2	15	3	3	1	2	3	2	3	2	3	2	3	2	2	2	2	3	2	2	2	3	3	3	2	3	4	3	3	3	3
177	2	1	19	2	30	3	3	3	2	3	2	3	3	2	2	3	3	2	2	3	3	3	3	2	3	3	3	2	3	3	3	4	3	4
178	1	1	19	4	75	5	5	5	5	5	5	4	5	5	4	5	5	4	4	3	3	2	4	4	4	4	4	4	4	5	5	5	5	3
179	1	1	19	2	30	4	4	4	4	4	4	4	5	4	5	4	5	4	5	4	4	5	4	3	4	2	4	3	4	5	3	3	4	1
180	1	1	19	5	35	4	5	4	4	5	4	2	2	4	2	3	5	5	4	3	5	3	4	3	3	3	3	1	3	5	2	3	4	1
181	1	1	19	1	45	5	5	5	5	5	5	5	5	5	5	5	5	5	5	5	5	5	5	5	5	4	5	3	3	1	1	1	2	1
182	2	1	19	1	60	2	2	1	2	1	2	2	2	2	2	1	3	3	2	3	3	2	3	4	3	2	2	1	2	5	3	2	3	1
183	1	1	19	5	45	3	3	3	2	2	3	3	2	4	2	4	5	2	3	2	2	2	2	3	3	3	4	2	3	4	3	2	3	2
184	2	1	19	2	30	1	2	1	1	2	2	1	1	2	1	1	2	2	1	1	2	1	2	1	2	2	1	2	2	4	3	3	4	3
185	1	1	19	7	90	5	5	4	5	5	5	5	5	4	5	4	5	4	3	3	4	3	4	4	4	4	4	3	4	5	3	3	4	4
186	1	1	19	5	45	3	4	2	3	2	3	3	2	3	2	1	5	1	1	1	1	1	1	1	1	3	1	2	1	3	3	2	5	1
187	1	1	19	7	35	3	4	3	3	4	3	3	3	4	3	4	5	4	4	4	4	4	4	3	3	4	3	1	4	3	1	3	4	3
188	2	1	19	2	60	4	4	3	4	5	4	3	3	3	3	4	4	5	5	3	5	4	4	4	4	5	4	3	4	4	3	4	4	4
189	1	2	20	1	120	2	4	1	2	2	1	1	1	1	2	2	2	4	3	3	4	4	5	5	4	4	3	4	4	5	4	4	3	4
190	1	1	20	1	10	3	3	4	4	4	3	4	3	4	4	3	4	2	3	4	3	3	3	2	3	3	3	4	4	2	3	5	4	5
191	1	3	20	3	60	1	3	1	1	2	2	2	1	2	1	2	3	3	2	2	3	4	4	4	3	1	3	1	2	4	3	5	4	2
192	2	1	19	2	120	3	4	3	3	3	2	2	3	3	3	4	1	4	4	4	4	4	4	4	4	3	3	1	1	4	4	4	4	4
193	1	1	19	4	120	3	3	3	3	3	3	3	3	3	3	3	4	4	4	4	4	4	4	4	4	4	4	3	3	3	3	3	3	3
194	1	2	21	2	60	3	4	5	3	4	3	2	2	1	1	4	5	4	3	4	3	4	3	4	4	3	4	4	2	5	3	5	3	3
195	2	1	21	1	15	3	5	3	5	3	3	3	3	3	3	3	3	4	3	3	4	4	4	4	4	4	4	4	4	4	4	4	4	4
196	1	1	22	4	90	3	3	2	3	3	3	3	3	4	4	2	3	4	4	2	5	4	5	4	4	4	5	2	3	4	3	3	2	1
197	1	1	22	3	90	3	4	3	3	3	3	2	2	3	3	1	2	4	4	3	4	4	4	4	4	4	4	1	3	5	4	4	4	4
198	2	1	22	1	30	1	1	1	2	3	1	1	1	1	1	1	3	4	2	3	3	3	2	1	1	2	1	2	3	5	3	3	2	2
199	2	1	22	1	30	2	3	2	3	1	2	1	1	1	1	1	1	3	3	3	3	2	4	2	2	2	3	1	2	2	2	2	3	2
200	1	1	22	4	60	3	4	3	3	3	4	3	3	3	3	3	4	4	4	4	4	2	4	2	3	3	4	3	3	3	3	3	4	4
201	1	1	22	7	60	3	4	3	3	2	3	3	3	3	3	3	3	1	1	1	1	1	1	1	1	1	1	1	1	4	4	3	3	3
202	2	2	19	1	30	3	4	3	3	2	3	3	2	2	2	2	3	2	3	2	2	2	2	2	2	2	2	2	2	4	2	4	3	2
203	2	4	19	2	90	2	3	3	3	4	3	1	1	2	1	3	1	3	3	2	4	4	4	2	2	2	2	1	1	3	4	3	4	3
204	1	2	19	1	30	2	2	2	2	2	3	3	2	2	3	1	3	3	2	3	3	4	3	4	4	3	4	1	2	2	2	4	3	1
205	2	2	19	2	120	2	4	3	3	2	3	3	2	2	2	2	2	4	4	2	4	4	5	4	4	4	5	2	2	5	3	5	5	2
206	1	2	19	2	120	2	4	2	3	3	2	3	2	2	2	2	2	4	3	2	4	3	4	4	3	3	3	3	2	4	3	4	4	2
207	1	1	21	2	30	4	4	3	5	3	5	3	4	4	4	3	4	4	4	4	3	3	3	4	3	2	3	3	3	4	4	3	2	2
208	1	1	19	3	60	4	4	4	4	3	4	4	4	4	4	4	4	3	3	3	3	3	3	3	4	4	4	5	4	4	4	4	4	4
209	1	1	21	3	15	3	4	3	3	3	3	3	3	3	3	3	3	3	3	3	3	3	3	4	5	4	4	4	3	5	4	4	5	5
210	1	1	21	1	30	2	3	3	4	3	4	3	4	3	2	4	2	2	2	2	2	2	2	2	2	2	2	2	2	4	4	4	4	3
211	1	1	20	4	90	2	3	2	4	2	2	2	2	2	2	2	2	4	2	3	3	3	3	3	3	3	3	3	2	3	4	4	4	4
212	2	1	19	1	120	2	4	2	4	2	4	3	3	1	2	2	3	3	3	4	3	3	3	4	4	2	2	1	2	4	3	4	4	3
213	1	4	23	3	90	4	5	4	4	4	4	5	5	4	4	4	5	4	5	5	5	5	4	3	4	5	4	4	4	4	2	2	4	1
214	1	2	20	3	10	2	2	1	2	3	3	2	1	3	1	3	3	3	3	2	3	1	2	3	2	3	3	2	2	4	3	5	5	3
215	2	1	24	1	15	2	3	2	2	3	2	3	3	3	2	2	3	3	3	4	3	2	3	3	2	3	2	2	3	2	3	3	2	3
216	2	1	19	2	60	4	3	2	3	4	4	4	3	3	4	4	3	3	4	4	3	5	5	4	4	3	3	2	3	3	4	3	3	2
217	2	1	20	1	145	3	2	2	2	3	2	2	3	2	3	3	3	4	4	4	4	3	2	3	3	4	3	2	3	3	2	3	2	1
218	2	1	20	2	90	3	5	5	4	3	4	2	5	5	5	5	5	3	3	3	3	3	3	3	3	3	3	3	3	2	2	3	2	3
219	1	1	20	5	100	3	5	3	4	2	4	4	4	4	4	3	2	5	5	5	4	4	4	4	4	4	4	4	2	4	4	4	4	3
220	1	1	20	3	20	1	1	1	1	1	1	1	1	1	1	1	1	1	1	1	1	1	1	1	1	1	1	1	1	1	1	1	1	1
221	1	1	21	4	60	3	4	2	3	3	3	2	3	3	3	2	2	4	3	2	4	4	3	4	4	2	3	2	3	3	3	3	4	2
222	1	1	21	7	60	1	3	1	2	3	2	1	1	1	1	1	1	4	4	4	4	5	4	4	4	4	4	1	2	3	4	2	4	3
223	1	1	20	5	30	2	3	2	3	4	2	3	1	1	1	2	2	3	4	2	4	4	3	4	4	3	3	2	4	3	3	2	2	1
224	1	1	20	4	90	3	4	3	4	3	4	4	3	4	4	3	4	3	4	3	3	3	4	4	4	4	4	4	4	3	4	4	3	4
225	2	1	20	1	30	3	4	3	3	4	4	3	3	3	4	4	4	3	4	2	4	4	4	4	3	3	4	3	2	4	3	4	4	4
226	2	3	20	1	60	3	4	4	3	3	3	4	3	3	2	3	4	3	4	5	4	4	3	3	3	3	3	3	4	4	4	5	5	4
227	1	1	21	2	90	3	5	3	3	1	3	3	4	2	4	3	3	4	4	4	4	3	3	4	4	3	4	2	2	3	4	4	5	1
228	2	1	19	1	10	4	4	4	4	4	4	4	4	4	4	4	4	4	4	4	4	4	4	4	4	4	4	4	4	4	4	4	4	4
229	2	1	19	1	60	1	1	1	1	1	1	1	1	1	1	1	1	3	4	4	4	4	3	3	4	4	4	4	3	3	3	3	3	3
230	1	1	22	7	120	2	5	2	3	5	4	5	2	2	2	4	5	4	4	4	5	5	5	5	4	4	4	4	5	4	2	4	4	2
231	1	1	24	7	60	3	4	2	4	4	3	4	3	2	3	2	3	4	5	4	5	5	4	4	5	4	5	4	4	4	3	3	4	2
232	1	1	20	3	120	1	2	1	2	1	1	2	1	1	2	1	1	5	4	5	5	4	5	4	4	5	5	4	5	4	3	5	4	5
233	2	1	22	1	60	5	5	5	5	5	5	5	5	5	5	5	3	4	4	4	4	4	4	4	4	4	4	4	4	5	5	5	5	5
234	1	1	24	4	80	3	1	3	2	4	3	1	3	3	4	3	1	4	2	4	3	4	1	3	2	2	4	4	1	2	4	3	1	4
235	1	1	23	4	180	5	3	5	3	2	1	1	5	3	4	5	2	4	2	5	2	4	2	4	1	5	5	5	3	4	2	3	1	3
236	1	1	24	4	80	5	3	5	2	1	3	1	5	3	2	1	4	5	3	4	1	3	5	2	3	2	1	3	1	2	1	3	4	5
237	2	1	18	2	60	5	2	5	3	5	4	5	4	2	3	5	2	2	3	2	5	2	4	2	5	3	2	1	3	3	2	4	1	2
238	2	1	19	2	20	5	2	5	1	5	3	2	5	3	1	4	2	2	5	2	4	2	5	3	1	4	3	1	4	2	1	4	2	5
239	2	1	18	2	60	3	5	2	5	3	5	2	3	2	5	3	1	2	5	3	1	4	3	1	3	1	3	2	5	3	2	5	2	1
240	1	1	19	2	60	2	5	3	1	5	3	5	3	5	3	5	2	2	5	3	2	3	2	5	3	2	5	4	2	3	3	2	1	5
241	2	1	20	1	30	3	3	1	3	2	3	2	3	2	1	2	1	3	3	2	2	1	2	1	1	3	3	3	3	3	3	2	3	1
242	2	1	19	2	60	2	3	3	3	2	3	3	3	3	3	3	3	3	3	3	2	3	3	3	3	3	3	3	3	3	3	3	3	3
243	2	1	19	2	90	2	4	2	2	4	2	2	2	2	2	2	3	2	4	2	2	2	2	2	2	2	2	2	3	4	2	4	4	3
244	1	1	24	3	90	2	3	2	2	3	2	2	2	2	2	2	3	4	3	2	3	3	3	3	3	3	3	3	3	5	5	5	5	3
245	1	1	19	4	60	2	4	3	3	4	3	3	3	3	3	3	3	4	4	4	4	4	4	4	4	3	4	3	4	4	4	4	4	4
246	1	1	19	6	70	4	3	4	4	4	3	5	4	2	3	4	3	4	3	4	2	3	4	4	3	4	3	3	3	3	4	3	3	4
247	2	1	19	2	60	2	3	1	2	2	5	4	4	1	5	1	1	4	4	1	1	4	4	4	4	1	4	1	1	1	1	1	1	1
248	2	1	20	2	15	2	4	2	2	3	2	2	2	2	2	2	3	3	3	2	3	4	3	3	4	3	3	3	2	3	2	4	4	4
249	1	3	22	3	90	3	2	4	4	4	3	4	5	4	3	4	4	4	4	4	4	4	5	4	5	5	5	4	5	5	3	5	4	4
250	1	1	23	4	60	3	3	3	3	3	3	3	3	3	3	3	3	5	5	5	5	4	5	4	5	4	5	4	5	4	4	4	4	4
251	1	1	22	3	45	3	3	3	4	4	3	3	3	4	3	3	3	5	5	4	4	5	4	4	5	4	4	4	5	5	5	5	5	5
252	2	1	19	2	60	3	3	3	2	3	3	3	2	2	2	3	3	3	3	4	4	3	3	3	3	4	3	4	3	4	4	4	4	3
253	1	1	20	2	45	3	4	4	4	3	3	3	4	4	3	3	4	4	4	4	3	4	4	4	5	4	4	5	5	4	4	3	4	3
254	2	1	20	2	20	4	4	3	5	4	4	4	4	4	4	4	4	4	4	4	4	4	5	4	4	4	4	4	4	4	4	4	4	4
255	2	1	20	2	60	3	4	3	5	5	3	4	4	4	3	4	5	4	3	4	4	4	4	5	4	4	4	4	5	3	4	4	4	4
256	2	1	20	1	45	3	4	4	3	4	4	4	3	4	3	4	3	3	3	3	4	3	4	4	3	4	3	4	4	3	3	3	4	3
257	2	1	20	2	30	3	3	3	4	4	3	3	4	4	3	3	4	4	4	4	3	4	4	3	4	3	4	4	3	3	3	3	3	3
258	2	1	19	2	15	3	3	3	3	3	3	3	3	3	3	3	3	4	3	4	5	5	3	2	3	3	4	3	3	4	4	3	3	5
259	2	1	20	2	120	3	4	3	4	2	4	2	3	4	4	3	5	5	4	3	4	3	3	3	4	4	5	5	5	5	5	4	5	3
260	2	1	18	2	60	2	5	1	3	2	2	2	1	3	2	4	5	5	4	4	4	4	5	5	5	4	4	3	3	5	5	5	3	4
261	2	1	20	1	45	3	3	3	3	2	2	3	3	3	3	2	1	4	4	4	4	4	5	5	5	5	5	5	2	5	5	5	5	4
262	2	1	19	2	90	3	3	2	4	3	3	1	1	1	1	1	1	3	2	1	3	1	2	2	1	2	1	1	1	3	2	3	1	1
263	2	1	18	1	30	2	4	2	2	3	2	2	2	2	2	2	1	3	3	2	3	4	4	2	2	2	4	2	2	3	3	3	3	3
264	2	1	19	1	30	4	3	4	5	3	4	5	4	3	4	5	4	3	4	4	4	3	5	4	4	4	3	4	5	3	4	5	4	3
265	2	1	19	2	45	2	5	3	4	4	4	3	3	3	3	3	3	5	5	3	4	4	5	5	4	4	4	4	4	5	5	4	5	4
266	2	1	19	2	20	2	2	1	2	1	2	1	1	1	1	1	1	2	1	1	2	1	2	2	3	3	3	2	1	4	3	3	4	2
267	2	1	19	1	40	2	2	1	2	1	1	1	1	1	1	1	2	2	3	1	3	3	3	3	2	2	3	1	1	4	4	3	4	1
268	2	1	19	1	30	2	2	2	2	3	3	1	3	1	3	4	1	5	4	4	4	4	4	4	5	4	5	4	3	5	4	5	5	4
269	2	1	20	2	30	2	2	3	3	3	3	3	3	3	4	3	4	4	4	2	4	4	4	3	4	4	3	4	3	4	3	3	4	4
270	2	1	20	1	45	3	2	3	3	3	3	3	2	2	2	3	2	3	3	3	3	2	2	3	2	2	2	2	2	3	2	3	4	3
271	2	1	20	2	30	3	3	3	3	3	2	3	2	3	2	2	4	4	4	3	3	4	4	3	3	4	4	4	4	4	4	4	4	4
272	2	1	24	1	30	2	2	2	3	3	3	2	3	2	2	3	3	1	1	1	2	1	2	1	1	1	1	1	2	3	2	2	3	2
273	1	1	21	3	60	3	4	3	3	4	3	4	3	3	3	3	4	3	5	5	5	5	5	5	5	4	5	4	2	4	3	3	4	4
274	1	1	21	2	30	1	1	1	1	1	1	1	1	1	1	1	1	2	1	1	2	1	1	2	1	3	1	1	3	3	3	3	3	1
275	1	1	21	2	60	2	3	2	2	3	3	2	1	1	1	1	4	1	3	4	4	4	3	4	4	3	3	2	2	3	2	3	3	4
276	1	1	24	2	15	3	3	3	4	4	4	3	3	4	3	2	3	3	3	3	4	4	4	4	3	4	4	4	3	5	3	5	5	5
277	2	2	19	2	30	4	4	4	4	4	4	4	5	4	4	4	4	3	3	2	3	3	2	3	2	3	3	3	3	3	4	4	4	3
278	2	1	20	2	120	3	4	3	4	2	4	2	3	4	4	3	5	5	4	3	4	3	3	3	4	4	5	5	5	5	5	4	5	3
279	2	1	20	1	45	3	3	3	3	2	2	3	3	3	3	2	1	4	4	4	4	4	5	5	5	5	5	5	2	5	5	5	5	4
280	2	1	18	2	60	2	5	1	3	2	2	2	1	3	2	4	5	5	4	4	4	4	4	5	5	5	4	3	3	5	5	5	3	4
281	2	1	20	1	60	4	4	4	4	4	4	5	4	5	4	4	4	5	5	5	5	4	4	5	5	5	4	4	3	5	5	5	5	5
282	1	1	21	4	60	3	3	3	3	4	3	3	4	4	3	3	4	3	3	3	3	4	4	3	4	3	3	4	3	4	4	4	3	2
283	2	1	20	1	30	4	4	4	4	4	3	3	4	4	3	3	3	5	5	5	5	5	5	4	4	4	4	5	5	5	5	5	5	3
284	1	1	21	7	90	3	4	4	4	3	5	4	4	4	3	4	5	3	4	4	5	3	5	3	4	5	3	4	3	4	5	4	5	4
285	2	1	19	2	30	3	4	3	4	3	3	4	4	3	3	4	4	4	5	3	3	4	3	4	4	3	2	3	5	4	3	4	4	3
286	2	1	19	2	30	4	3	3	4	5	4	3	4	4	4	3	4	4	5	4	3	4	4	5	3	3	4	3	4	5	4	5	4	4
287	1	1	19	6	90	5	4	4	5	3	4	4	3	4	3	4	4	4	3	4	3	3	4	5	4	5	4	3	4	4	3	5	4	4
288	1	1	21	2	30	3	4	3	4	3	3	3	3	3	2	2	4	4	4	3	4	4	4	3	3	3	4	2	4	3	3	4	4	3
289	1	1	22	4	15	2	2	2	2	3	2	2	2	3	2	3	4	3	2	2	3	4	2	2	3	3	3	2	2	3	2	3	4	4
290	1	1	20	2	60	1	2	2	2	2	2	2	1	2	1	2	2	1	2	2	2	2	3	2	2	2	2	2	3	1	1	1	1	1
291	1	2	19	4	50	2	3	3	3	4	3	3	4	2	3	3	3	4	3	3	4	3	3	4	3	4	3	4	4	4	4	3	3	3
292	1	1	21	3	60	1	5	2	3	3	3	3	2	3	2	3	4	3	4	2	3	4	3	3	2	2	3	3	4	3	2	3	2	2
293	2	1	19	2	35	2	2	2	2	2	2	2	2	2	2	2	4	4	4	4	4	4	4	4	2	1	2	2	2	5	5	5	5	5
294	2	1	20	2	20	3	1	1	3	2	3	2	1	1	1	2	3	4	3	3	5	5	3	3	4	4	5	2	4	5	4	3	5	3
295	2	1	19	2	60	2	3	3	3	2	3	3	3	3	3	3	3	3	3	3	2	3	3	3	3	3	3	3	3	3	3	3	3	3
296	1	1	21	2	60	3	4	3	3	3	3	2	2	2	3	3	3	2	2	2	3	3	1	1	3	2	3	1	4	4	2	2	3	1
297	2	1	20	1	30	3	5	4	3	3	4	3	3	3	3	3	3	5	5	5	5	5	4	5	4	5	4	4	5	5	5	5	5	5
298	1	2	20	3	40	3	4	4	4	4	3	3	3	3	4	4	4	4	4	3	4	4	4	4	4	4	4	3	2	4	4	4	4	1
299	2	1	19	1	30	3	4	2	2	3	4	1	3	2	2	2	1	3	2	2	3	5	4	3	3	3	3	2	2	3	2	3	3	2
300	2	1	19	1	45	1	2	1	2	3	2	1	1	1	1	1	2	3	2	1	3	3	3	3	4	3	3	1	1	4	4	4	4	4
301	2	1	19	1	45	1	2	1	2	3	3	2	2	3	2	2	2	3	2	2	3	2	3	3	2	3	2	2	2	4	4	4	4	4
302	2	1	19	2	40	1	5	1	1	3	1	2	1	2	1	3	4	3	3	2	2	5	3	3	2	3	3	2	2	5	2	4	5	2
303	2	1	19	1	40	1	1	1	1	1	1	1	1	1	1	1	1	1	1	1	1	1	5	4	3	1	1	3	3	3	3	3	3	2
304	2	1	19	2	30	1	1	1	1	1	1	1	1	1	1	1	1	2	1	1	1	1	1	1	1	1	1	1	1	5	2	4	4	3
305	1	2	21	2	60	3	4	5	3	4	3	2	2	1	1	4	5	4	3	4	3	4	3	4	4	3	4	4	2	5	3	5	3	3
306	2	1	20	2	30	4	4	4	4	3	3	4	3	4	3	4	3	4	3	4	4	3	4	3	3	4	3	3	4	3	3	3	3	3
307	2	1	19	2	180	3	4	3	3	5	4	4	3	3	3	5	4	5	5	4	5	5	4	4	5	4	5	5	4	3	3	3	4	4
308	2	1	19	1	60	1	1	1	1	1	1	1	1	1	1	1	1	3	2	1	1	2	2	1	1	1	1	1	1	3	3	3	3	2
309	2	1	20	2	45	1	2	1	2	2	2	1	1	1	2	2	2	3	2	3	3	2	3	3	4	3	2	3	3	3	2	2	2	1
310	1	4	18	2	90	2	2	2	2	2	2	2	2	2	2	2	2	4	3	4	3	3	2	3	2	3	2	3	3	3	2	2	2	1
311	1	1	20	7	60	2	4	2	2	1	3	4	2	1	2	1	3	4	3	2	4	1	4	5	2	2	4	3	3	3	2	2	3	2
312	2	3	19	2	120	3	4	3	3	3	4	4	5	3	4	4	3	5	5	4	4	5	4	3	4	5	4	5	5	5	5	4	5	4
313	2	1	19	1	180	5	5	4	4	4	3	4	5	4	4	4	3	5	5	5	4	4	3	4	4	5	4	5	4	5	5	5	5	5
314	2	1	19	1	60	3	4	4	4	4	3	3	3	3	4	4	3	3	3	3	3	3	3	3	3	3	3	3	3	4	4	4	4	4
315	2	1	18	1	30	3	3	3	3	3	3	3	3	3	2	2	2	3	3	3	3	3	3	3	3	2	2	3	3	3	3	3	3	3
316	1	1	20	4	60	3	4	3	3	5	4	4	3	3	3	4	4	4	4	3	5	4	4	4	4	4	4	3	4	5	4	4	4	4
317	2	1	20	0	0	2	2	2	2	2	2	2	2	2	2	2	2	3	3	4	2	3	4	4	3	4	3	2	3	4	2	4	4	3
318	2	1	19	2	90	3	5	3	3	3	3	4	4	4	4	4	3	5	5	3	4	5	3	5	5	3	5	3	3	5	3	5	5	5
319	2	1	20	2	120	2	3	2	3	1	3	2	2	2	2	2	2	3	3	3	3	2	3	3	3	3	2	3	2	3	2	2	3	1
320	2	1	18	1	60	2	2	3	3	2	2	3	2	3	3	3	2	3	4	3	4	2	5	2	3	3	4	3	2	3	3	3	2	2
321	2	1	19	2	60	2	4	2	3	2	2	2	2	2	3	3	3	4	4	3	2	2	3	3	2	2	3	2	2	2	3	3	4	2
322	2	1	19	2	60	3	3	3	3	3	3	3	3	3	3	3	3	2	2	1	2	2	2	2	2	2	2	1	2	2	2	3	3	3
323	2	1	19	2	120	3	3	4	4	4	4	3	3	3	4	4	4	4	4	3	3	3	4	4	3	3	4	4	3	3	2	2	4	2
324	1	1	19	5	60	4	4	3	4	4	3	3	4	4	4	3	3	3	3	3	2	4	3	2	4	3	2	4	2	3	3	4	2	3
325	1	1	19	2	60	3	3	3	4	4	4	3	3	3	4	3	3	4	4	4	4	5	4	4	4	4	4	3	3	4	4	4	4	3
326	2	1	19	2	90	3	3	2	4	4	2	2	3	4	2	3	4	3	3	4	4	4	4	3	3	3	3	3	4	3	3	4	4	4
327	1	1	21	1	60	4	4	3	3	5	5	5	3	3	3	5	5	4	3	3	3	2	5	5	4	4	4	1	4	4	3	3	3	2
328	2	1	19	2	30	3	4	2	3	4	3	2	3	3	2	3	4	4	4	3	4	5	2	2	3	3	3	2	4	5	4	3	3	2
329	1	1	23	4	30	1	4	1	1	1	5	1	1	4	1	1	5	1	4	1	1	1	1	1	1	1	1	1	5	5	5	5	5	1
330	2	1	19	2	60	2	3	1	2	2	5	4	4	1	5	1	1	4	4	1	1	4	4	4	4	1	4	1	1	1	1	1	1	1
331	1	1	21	5	120	2	5	2	3	4	5	3	2	1	4	1	5	5	5	5	5	5	3	4	4	4	4	1	1	1	1	1	5	3
332	1	1	20	2	90	2	2	1	2	2	2	2	2	2	2	2	3	2	2	1	2	2	2	2	2	2	2	2	3	1	1	1	1	1
333	1	1	20	5	90	4	4	3	4	2	3	2	2	3	2	2	3	4	3	3	3	2	3	2	2	2	2	2	2	3	3	4	4	2

334	1	1	21	1	150	4	4	4	4	3	4	3	4	4	4	4	4	4	4	4	4	3	4	4	4	4	4	4	4	4	3	3	3	3
335	1	1	20	5	45	3	3	3	4	3	4	4	3	4	3	4	3	3	4	4	3	4	3	4	3	4	3	3	4	3	4	3	3	4
336	2	3	20	1	30	3	4	3	5	3	2	3	4	5	3	4	5	3	4	3	5	4	3	4	5	4	3	4	4	3	3	4	3	4
337	1	1	23	4	90	3	4	3	3	3	3	4	3	3	4	4	5	5	5	5	4	4	4	4	5	4	5	5	5	4	5	4	5	4
338	2	4	21	1	30	2	3	3	3	3	3	3	3	3	3	3	4	3	4	3	4	4	4	4	4	4	4	3	4	5	5	5	5	3
339	2	1	21	1	30	2	3	2	3	3	3	2	3	2	3	3	3	3	3	3	4	3	3	3	4	3	3	4	4	4	4	4	4	4
340	2	4	19	1	60	4	5	3	4	3	4	2	3	2	3	1	5	3	4	3	4	1	3	2	3	1	1	1	3	3	3	5	4	3
341	2	1	19	1	60	2	3	2	3	2	2	1	1	1	1	1	2	4	4	3	3	3	4	3	4	3	3	3	2	5	4	4	3	4
342	1	2	23	3	90	1	1	1	1	2	1	2	1	1	1	3	1	4	4	3	4	3	4	4	3	2	4	2	2	3	2	3	4	1
343	2	1	19	2	30	4	4	2	4	2	3	3	3	2	3	2	3	4	3	2	4	4	4	3	3	3	4	2	3	3	2	4	3	2
344	1	4	24	3	50	1	1	1	1	1	1	1	1	1	1	1	2	5	4	4	4	4	4	4	4	4	4	3	5	4	3	4	4	2
345	1	1	24	2	90	2	4	1	2	2	2	2	2	2	2	2	2	4	3	2	3	3	3	3	2	2	2	2	2	5	5	5	5	2
346	1	1	19	3	75	2	4	2	3	3	4	4	3	3	4	3	4	5	4	3	3	5	4	5	4	4	5	3	3	5	4	4	4	2
347	1	1	19	4	60	4	4	3	4	4	2	3	3	4	4	4	4	4	4	4	4	5	5	3	3	4	4	3	5	5	5	4	4	3
348	1	1	22	3	60	3	3	2	3	2	3	1	4	2	2	2	1	3	2	3	2	3	2	2	2	3	3	3	1	5	4	3	4	3
349	1	1	19	1	120	1	2	1	1	3	3	3	3	3	3	3	3	1	3	2	2	2	3	3	2	1	1	2	3	2	2	2	2	2
350	1	1	19	2	20	4	4	4	4	4	3	3	2	3	4	3	5	5	4	4	5	4	3	4	5	4	3	4	5	3	4	3	4	5
351	1	1	20	4	60	2	4	2	2	3	2	2	2	2	2	2	3	4	3	3	3	3	4	3	3	3	3	2	3	4	3	2	3	2
352	2	1	19	2	60	2	3	2	3	2	3	3	3	2	3	3	3	4	3	3	4	4	4	3	4	3	4	3	2	5	4	4	5	3
353	1	1	20	2	#NULL!	2	4	2	3	3	3	2	3	2	3	2	3	4	4	2	3	3	4	4	4	3	3	4	3	4	4	3	4	3
354	2	1	19	1	90	4	3	2	2	3	2	2	2	2	2	3	4	4	3	3	3	3	2	2	2	2	2	3	4	3	3	4	3	3
355	2	1	19	2	40	4	3	2	3	3	2	2	2	2	2	3	4	3	2	2	3	2	2	2	2	2	2	3	4	3	3	4	4	4
356	2	1	19	2	30	4	4	3	4	5	4	4	3	4	4	4	4	4	4	5	4	3	3	4	4	4	4	4	3	4	3	4	4	1
357	2	1	19	1	90	4	3	2	3	2	3	2	2	2	1	2	4	4	3	2	3	3	3	2	2	3	2	2	4	3	2	3	3	2
358	2	1	19	0	0	1	2	1	1	3	1	1	1	1	1	2	2	3	2	1	3	2	3	1	1	1	1	1	3	5	4	5	4	3
359	2	1	18	1	60	1	2	1	2	3	2	1	1	2	2	1	3	3	3	2	4	4	4	4	2	2	2	1	3	3	4	3	4	3
360	1	1	21	2	120	1	1	1	1	1	1	2	2	1	2	2	1	4	4	4	4	2	3	4	4	4	4	3	2	3	3	3	4	3
361	1	1	23	4	60	4	3	3	3	4	2	3	3	3	4	4	4	3	3	4	4	4	3	4	4	4	5	5	4	4	4	5	5	5
362	2	1	19	2	90	4	4	5	5	5	4	4	5	5	5	5	4	4	3	3	5	3	5	3	4	4	4	3	3	5	5	5	3	2
363	2	1	19	2	60	1	1	1	1	1	1	3	1	3	1	1	1	4	1	3	4	2	3	3	3	2	2	2	1	4	3	4	4	1
364	2	1	19	1	15	1	2	1	2	1	1	1	2	1	2	1	2	3	2	1	3	2	3	2	3	1	4	1	1	5	5	5	3	2
365	2	1	20	1	60	1	3	1	1	1	1	1	1	1	1	1	1	5	4	4	5	4	5	5	4	5	5	4	5	4	4	5	3	3
366	2	1	19	2	90	2	1	1	2	1	3	1	1	1	1	1	1	3	3	2	3	4	3	1	2	1	1	1	1	5	4	5	3	5
367	2	1	19	1	60	4	4	2	5	2	3	2	2	2	2	1	1	4	4	2	4	2	3	3	3	3	3	2	2	2	2	2	4	2
368	2	1	19	2	30	2	5	2	2	3	3	2	2	3	2	1	4	4	5	5	5	4	3	3	4	3	5	2	3	5	3	5	5	4
369	2	1	19	2	120	4	4	4	4	4	4	4	3	3	3	3	3	4	4	4	4	4	4	4	4	4	4	4	4	4	4	2	2	1
370	1	1	19	2	90	3	4	2	3	2	2	2	2	2	2	2	4	4	3	3	4	4	3	3	2	1	3	2	3	3	4	4	3	3
371	1	1	19	1	15	2	2	1	2	2	3	1	2	2	2	2	2	3	3	1	2	2	3	3	3	2	2	1	1	3	3	4	3	3
372	2	1	19	2	30	2	3	2	3	5	2	2	3	3	3	2	1	3	4	2	3	2	3	3	2	2	2	2	3	5	3	4	4	2
373	2	1	19	2	60	3	4	2	3	1	1	1	2	2	2	2	2	3	2	2	3	1	3	1	3	1	1	1	1	3	3	3	3	3
374	1	1	18	1	180	4	4	4	4	4	4	4	4	3	3	3	3	4	3	4	3	4	3	4	3	4	3	4	3	4	4	4	3	3
375	1	1	19	3	180	4	3	5	4	4	5	3	4	3	3	4	5	4	3	3	5	5	4	3	3	3	4	5	5	4	4	3	5	5
376	1	1	20	3	180	4	3	3	4	4	3	3	3	4	4	4	4	4	4	4	4	4	4	4	4	4	4	4	4	3	3	3	3	3
377	2	1	19	2	30	3	4	1	3	3	3	4	2	1	1	1	1	3	3	2	3	3	3	3	3	3	3	2	2	4	4	4	4	4
378	1	1	23	7	60	2	2	2	3	3	3	3	3	3	4	4	4	2	4	2	2	2	2	2	2	2	2	2	2	3	4	2	3	4
379	2	1	19	1	60	2	3	2	2	2	2	3	2	3	2	2	3	2	2	2	2	3	2	3	2	3	2	2	2	4	4	4	4	1
380	2	1	19	1	30	3	2	2	2	2	3	3	2	3	2	3	2	3	3	2	2	2	3	3	2	2	2	2	2	3	3	3	4	3
381	2	1	21	1	30	3	3	3	4	2	4	4	4	4	3	3	3	3	3	3	3	3	3	3	3	3	3	3	3	4	4	4	4	4
382	2	4	19	1	60	4	2	2	3	2	3	3	3	4	2	3	4	3	4	2	3	1	2	2	2	3	1	1	2	4	3	4	4	3
383	1	3	22	2	30	2	2	2	3	2	2	2	2	3	2	3	3	3	3	3	3	2	2	2	2	2	2	3	3	4	4	4	3	3
384	2	4	20	1	90	2	2	2	2	2	2	2	3	3	2	2	3	3	3	2	3	2	3	3	2	2	2	2	3	4	3	4	3	3
385	1	1	20	6	37	3	2	2	3	2	2	2	2	2	3	3	3	4	4	4	4	3	4	3	3	3	4	3	4	4	3	3	4	3
386	2	1	20	1	50	3	4	3	3	4	3	4	4	4	4	4	2	3	2	3	3	3	3	2	3	3	2	3	2	2	1	2	2	2
387	2	4	18	2	30	2	3	2	2	3	2	3	2	2	3	2	3	3	2	3	4	3	4	3	4	3	3	2	2	4	4	4	3	3
388	2	4	19	2	45	3	4	2	3	3	4	2	3	3	2	1	1	3	2	3	3	4	3	3	3	3	2	2	1	4	1	1	4	2
389	1	1	19	3	120	2	4	3	2	1	2	2	1	3	2	2	1	5	4	5	4	3	4	5	3	4	5	4	3	4	2	1	3	3
390	2	2	19	1	60	4	2	2	3	2	3	3	3	4	2	3	4	3	4	2	3	1	2	2	2	3	1	1	2	4	3	4	4	3
391	2	1	19	2	5	1	1	1	1	1	1	1	1	1	2	1	1	3	3	3	1	4	1	2	1	1	1	1	1	1	1	1	1	1
392	2	1	19	2	60	2	4	2	3	4	5	3	3	4	3	2	2	5	5	3	4	3	4	4	4	4	4	3	2	4	4	4	4	3
393	2	2	19	2	90	2	3	3	3	3	2	3	2	2	2	3	4	3	3	4	3	3	3	2	3	3	3	2	4	3	3	3	2	3
394	1	1	20	3	120	3	3	3	2	4	3	3	2	4	4	3	1	4	4	4	3	4	3	3	3	2	3	4	1	1	1	2	2	1
395	2	1	19	1	20	2	2	2	2	2	2	2	2	2	2	2	1	2	2	2	2	2	2	2	2	2	2	2	2	5	5	5	5	1
396	2	1	19	1	10	3	2	2	3	3	3	2	2	2	2	3	4	5	4	4	4	4	5	4	4	4	4	4	5	5	4	4	4	4
397	2	1	19	2	60	2	3	3	3	3	3	3	3	3	3	3	3	4	3	3	4	3	2	3	3	3	3	3	2	5	5	5	5	4
398	1	1	20	1	60	1	1	1	1	1	1	1	1	1	1	1	3	3	3	3	3	3	3	3	3	3	3	3	3	5	5	5	5	5
399	2	1	19	2	30	4	4	4	4	4	4	3	3	4	4	4	3	4	4	4	4	4	5	3	4	4	5	3	3	4	3	3	3	3
400	2	1	19	2	90	1	3	1	1	3	3	3	2	2	3	3	2	3	4	3	3	4	4	4	4	3	4	2	1	4	3	4	4	3
401	1	2	21	2	60	3	4	5	3	4	3	2	2	1	1	4	5	4	3	4	3	4	3	4	4	3	4	4	2	5	3	5	3	3
402	1	1	20	1	15	2	2	2	2	1	1	1	2	2	2	2	2	3	2	2	3	3	3	2	3	3	3	3	2	1	1	2	2	2
403	2	1	19	2	60	1	4	1	3	1	3	4	1	4	1	1	2	1	1	1	2	1	2	3	3	1	3	1	3	3	1	4	3	5
404	2	1	19	2	15	3	5	4	4	5	4	4	4	4	4	4	5	5	5	5	4	4	5	4	4	5	4	4	4	3	3	4	3	4
405	2	1	21	1	30	1	3	1	1	1	1	1	1	1	1	1	1	3	3	2	2	2	2	1	2	2	2	1	2	4	2	3	4	2
406	2	1	21	1	30	1	3	3	3	3	3	3	3	3	3	3	3	4	4	4	4	4	4	4	4	4	4	4	1	3	3	2	3	2
407	2	3	19	2	45	2	3	3	3	3	3	3	2	2	2	3	3	3	4	4	4	3	4	4	3	3	3	4	4	3	3	3	4	4
408	2	1	19	2	60	2	5	2	3	2	3	3	3	3	3	3	2	2	5	2	3	2	3	3	3	3	3	3	4	3	2	5	5	1
409	1	1	21	2	30	3	3	3	4	3	4	3	3	2	3	4	3	3	4	3	3	4	4	4	3	3	3	4	4	3	3	3	3	3
410	2	1	19	2	180	2	5	1	3	4	3	4	3	4	4	4	3	3	4	3	4	4	4	3	4	3	3	3	3	1	2	2	1	1
411	1	1	19	6	45	1	1	1	3	2	4	4	3	3	4	1	5	3	4	2	3	3	2	4	4	3	4	2	4	5	5	5	5	5
412	1	1	19	7	60	3	4	3	4	5	3	4	3	4	5	4	4	4	5	4	3	3	4	3	4	3	5	4	3	4	3	5	4	4
413	2	1	18	2	30	3	2	2	3	2	3	3	3	3	3	2	2	3	2	2	3	2	3	3	3	3	3	3	3	5	4	5	5	4
414	2	1	19	2	30	3	4	2	4	4	2	4	4	3	4	3	2	2	4	2	3	3	4	4	4	4	4	3	4	5	4	4	4	4
415	1	1	22	2	90	1	2	2	1	2	2	3	2	2	1	2	2	3	3	2	4	4	4	3	3	4	3	3	3	2	3	3	2	2
416	2	1	19	1	20	2	4	3	2	2	4	3	4	2	3	4	4	5	4	4	4	4	4	5	4	4	4	5	4	3	2	2	2	3
417	1	1	19	2	60	2	4	2	3	5	3	4	3	2	3	3	3	3	4	3	3	4	3	4	4	3	3	2	2	5	4	4	5	4
418	1	1	25	2	120	1	1	1	1	3	1	1	1	1	1	1	2	3	1	3	3	3	3	3	3	3	3	3	3	1	1	1	1	1
419	2	1	19	2	120	4	4	3	3	4	3	2	2	2	2	2	3	4	2	3	4	4	3	3	3	2	3	1	3	4	4	4	4	3
420	2	1	20	1	30	1	2	1	1	2	2	2	2	2	2	2	2	3	3	3	3	3	4	4	3	3	3	3	2	4	3	4	4	2
421	2	1	19	2	30	3	3	4	4	4	3	4	4	4	3	4	5	4	4	4	4	4	5	4	4	4	5	5	5	3	4	4	3	4
422	2	1	19	2	60	2	3	2	2	2	2	2	2	1	2	1	3	4	3	3	4	3	3	3	3	2	3	2	2	4	4	4	2	2
423	2	1	20	1	30	2	3	1	2	3	1	1	1	1	1	1	4	4	3	1	2	4	2	2	2	2	2	2	5	4	4	4	4	4
424	2	1	19	2	60	2	2	1	3	3	3	3	3	3	3	2	2	4	3	1	4	2	2	3	3	3	4	3	3	3	4	4	4	3
425	2	1	19	2	90	3	3	3	3	3	3	3	3	3	3	3	3	4	4	4	4	4	4	4	4	4	4	4	4	4	2	3	4	3
426	2	1	19	2	90	2	3	2	3	3	2	2	3	3	2	2	2	4	3	3	3	4	4	3	4	3	3	2	2	3	3	3	3	3
427	2	1	19	2	90	4	4	4	4	4	4	4	4	3	3	3	2	4	4	4	4	4	3	3	3	4	3	4	3	4	4	4	5	3
428	2	1	19	1	15	1	1	1	1	1	1	1	1	1	1	1	2	3	2	2	3	2	2	2	2	1	1	2	1	3	3	2	3	3
429	2	1	25	1	20	2	2	2	3	2	3	2	2	2	2	2	2	2	2	3	3	2	3	2	2	2	2	2	2	4	4	3	4	3
430	1	1	20	3	30	1	2	1	2	2	1	2	1	1	1	1	3	3	3	1	2	2	1	2	2	2	3	1	3	4	4	3	3	4
431	1	1	20	7	120	2	4	2	2	4	3	2	2	2	2	2	4	4	4	3	4	4	4	4	4	4	4	3	4	5	4	4	5	4
432	1	1	25	4	120	3	3	4	4	4	3	3	3	4	3	3	4	4	3	2	3	4	3	2	4	3	2	3	4	3	4	3	2	4
433	2	1	19	2	60	2	3	3	3	3	2	2	2	2	3	2	2	3	3	3	3	3	3	3	3	3	3	3	3	4	4	4	4	3
434	2	1	19	2	30	4	5	5	5	5	5	5	5	5	5	3	1	3	4	2	3	4	4	4	4	4	4	1	1	3	2	4	4	4
435	2	1	19	2	30	2	1	1	2	1	2	2	1	1	1	1	1	2	1	1	1	1	2	1	1	1	1	1	1	4	2	3	3	1
436	2	1	19	2	60	2	3	1	5	5	5	3	3	3	3	1	3	4	4	4	4	2	4	4	4	4	4	4	4	3	3	3	3	3
437	2	1	20	1	120	3	3	3	3	3	3	3	3	3	3	3	3	4	5	3	4	1	2	3	4	5	4	3	4	3	2	3	3	3
438	2	1	19	2	45	3	4	3	3	3	2	2	3	3	3	3	3	4	4	4	4	4	4	4	4	4	4	4	4	4	4	4	4	4
439	1	1	20	2	45	4	3	2	3	2	2	2	3	3	3	4	2	4	4	3	3	3	4	3	3	4	3	4	4	3	4	3	4	4
440	2	1	20	1	60	2	3	2	2	4	4	4	3	3	3	3	4	5	5	5	5	5	5	5	5	5	5	5	5	4	3	2	4	2
441	1	1	20	5	45	3	4	3	3	4	5	3	3	4	4	2	3	5	4	4	5	4	5	5	3	3	4	5	4	4	3	4	4	3
442	2	1	19	2	30	4	3	4	4	3	5	3	4	5	4	5	4	3	4	3	3	4	5	3	4	4	4	5	4	4	3	3	4	3
443	1	1	20	2	20	3	4	3	3	4	3	4	3	3	4	3	4	3	4	4	3	4	3	4	3	4	3	3	4	3	3	3	3	3
444	2	1	20	1	20	4	4	4	3	4	3	3	3	3	4	4	3	3	4	4	3	3	4	3	4	3	4	3	4	3	4	4	4	5
445	2	1	20	1	60	4	5	4	5	5	5	4	5	4	5	5	5	5	4	5	5	5	4	4	5	4	5	4	5	4	4	5	5	4
446	1	1	20	4	30	3	4	4	4	3	3	3	4	3	4	3	4	3	3	4	4	4	3	3	4	4	3	3	3	3	3	4	4	4
447	1	1	23	2	20	3	3	3	3	4	4	3	4	3	4	3	3	3	3	4	3	3	3	4	3	4	3	4	4	3	3	3	3	3
448	2	1	19	2	90	3	4	2	3	3	3	3	3	3	3	2	4	3	4	4	4	3	4	4	4	4	4	4	4	5	4	5	5	3
449	2	1	19	2	90	1	2	4	4	4	4	3	2	2	3	1	1	4	3	4	4	4	4	3	2	2	3	2	1	4	2	4	4	3
450	2	1	19	1	15	3	2	2	3	4	1	1	1	1	1	2	1	3	3	3	3	3	3	3	3	3	3	3	3	2	2	2	3	2
451	2	1	19	1	120	2	5	2	3	5	2	3	3	2	2	2	4	3	5	3	4	3	4	2	3	2	2	2	3	5	5	5	5	5
452	2	1	19	1	60	2	4	2	2	4	1	1	1	1	1	1	4	5	5	5	5	5	5	5	5	5	5	5	5	3	3	3	3	3
453	2	1	19	2	90	2	3	3	2	2	3	3	2	3	3	2	3	3	4	3	4	4	4	4	4	4	4	3	4	4	3	5	5	3
454	1	4	19	3	50	2	3	1	2	2	1	1	2	1	2	1	3	3	2	2	3	3	2	2	2	2	3	2	3	3	3	4	3	3
455	2	4	19	2	20	2	3	1	2	2	3	1	2	2	2	3	2	4	4	3	4	2	2	3	3	3	3	2	3	2	1	1	1	1
456	2	1	19	2	90	1	2	1	2	3	2	2	2	2	2	2	2	3	2	2	2	2	2	2	2	2	2	2	2	4	3	4	3	3
457	2	1	19	2	90	3	3	3	3	3	3	3	3	3	3	3	3	3	3	3	3	3	3	3	3	3	3	3	3	3	3	3	3	3
458	2	1	19	1	120	2	5	4	5	3	3	3	3	3	1	3	5	3	3	3	3	3	3	3	3	3	3	3	3	3	3	1	3	3
459	2	1	19	2	90	2	3	2	2	2	2	2	1	1	1	2	3	2	3	2	2	3	2	2	3	2	2	2	2	3	4	1	2	3
460	2	1	19	1	60	2	3	3	3	3	4	3	3	3	4	3	3	4	3	3	3	4	3	3	3	3	4	3	3	3	3	3	3	4
461	1	1	19	2	80	3	3	3	2	3	3	3	3	3	4	4	4	4	4	3	4	4	4	4	3	4	4	4	4	3	4	4	3	3
462	1	1	21	3	60	4	4	4	5	4	4	4	4	4	4	5	5	4	3	3	4	3	4	4	4	4	4	3	4	3	4	4	3	4
463	2	1	19	2	90	3	2	3	3	3	4	4	4	4	4	3	3	3	4	4	4	3	4	4	4	4	4	5	5	3	4	3	4	4
464	1	1	24	2	30	4	5	4	4	4	4	4	4	4	4	5	5	3	3	3	4	3	3	3	3	4	4	4	3	3	3	3	3	3
465	1	1	21	3	120	2	3	3	3	3	3	3	3	3	3	3	3	5	3	4	4	5	5	3	4	4	3	3	4	5	3	3	5	2
466	2	1	18	2	30	2	2	1	2	2	3	2	2	1	2	1	2	2	2	2	3	1	1	3	2	1	2	1	1	5	3	3	4	3
467	2	1	19	2	45	2	1	1	1	1	3	1	1	1	1	1	2	4	1	1	1	2	3	3	2	1	2	1	1	4	5	5	5	2
468	1	1	21	3	120	2	3	3	2	2	2	3	3	2	2	3	3	5	4	3	4	4	5	3	4	4	3	3	4	5	3	3	5	2
469	1	1	21	3	120	3	3	2	2	3	2	1	1	1	1	1	2	3	3	2	3	2	3	3	3	2	3	3	2	5	3	3	5	2
470	1	1	21	3	120	2	2	1	1	1	1	1	1	1	2	1	2	3	4	4	4	4	4	3	4	3	4	3	2	5	3	3	5	2
471	2	1	19	1	540	2	2	2	2	3	3	2	3	3	3	3	3	4	3	2	3	2	3	3	2	2	2	2	2	4	4	4	4	4
472	1	1	21	3	120	2	3	2	3	3	3	2	2	2	3	3	3	5	3	4	4	5	5	3	5	4	4	3	4	5	3	3	5	2
473	1	1	20	2	60	3	3	3	3	2	3	3	2	2	2	2	3	3	2	2	3	2	2	2	2	3	3	3	3	3	3	3	3	3
474	2	1	20	1	20	2	2	1	2	1	1	1	1	1	2	2	2	3	3	2	2	2	2	2	3	2	2	2	2	3	3	3	3	3
475	2	1	22	1	20	2	3	2	3	1	3	3	3	3	2	2	1	3	2	2	2	1	3	1	1	1	2	1	1	4	2	3	3	3
476	1	1	20	2	120	3	3	3	3	3	3	3	3	3	3	3	4	4	4	3	4	3	4	3	3	3	3	3	4	4	4	4	4	4
477	2	1	20	1	120	2	3	2	2	2	2	2	2	2	2	2	3	3	3	3	3	3	2	2	2	2	2	2	3	3	3	3	3	3
478	2	3	20	1	60	3	2	2	2	2	3	3	3	2	3	2	2	2	3	3	3	3	2	2	2	3	3	3	2	3	3	3	3	3
479	1	1	20	2	60	4	4	3	4	3	4	4	3	4	4	4	4	4	4	4	4	4	4	4	4	3	4	3	3	4	4	4	4	4
480	2	1	20	1	60	3	4	2	3	4	3	2	3	2	5	1	1	2	4	1	2	3	2	1	1	1	2	1	3	4	3	3	4	2
481	2	1	20	1	20	3	4	2	2	2	2	2	2	2	2	3	3	3	3	3	4	4	3	4	2	3	2	2	3	4	2	2	4	4
482	2	1	20	1	60	3	4	2	3	4	3	2	3	2	5	1	1	2	4	1	2	3	2	1	1	1	2	1	3	4	3	3	4	2
483	2	1	20	1	20	3	4	2	2	2	2	2	2	2	2	3	3	3	3	3	4	4	3	4	2	3	2	2	3	4	2	2	4	4
484	1	1	24	1	60	2	4	2	2	5	4	4	2	3	3	3	4	2	2	2	2	4	3	3	3	3	4	2	4	4	4	4	4	2
485	1	1	20	1	120	2	4	1	2	2	1	1	1	1	2	2	2	4	3	3	4	4	5	5	4	4	3	4	4	5	4	4	3	4
486	2	1	20	1	60	2	4	2	2	1	3	1	2	2	2	2	2	3	2	3	3	2	3	3	3	3	3	2	2	4	4	4	4	4
487	1	1	22	5	30	2	2	2	2	2	2	3	2	2	3	3	2	4	4	3	2	2	2	2	3	3	3	4	3	4	4	4	4	4
488	1	1	19	3	1	2	2	1	2	2	1	1	2	1	1	1	1	3	3	3	3	3	3	3	3	1	2	1	1	4	3	2	4	3
489	1	1	21	1	30	3	2	2	3	2	1	1	1	1	2	2	1	2	2	2	2	4	3	3	3	3	4	2	4	4	4	4	4	2
490	1	1	19	3	60	2	4	1	2	2	1	1	1	1	2	2	2	4	3	3	4	4	5	3	3	3	4	4	4	5	4	4	3	4
491	1	1	19	3	60	3	4	3	3	4	3	3	3	3	3	3	4	4	4	3	4	5	5	5	5	4	4	4	4	4	3	3	3	3
492	1	1	19	3	60	2	3	3	4	3	2	3	3	4	4	3	2	4	3	3	4	2	3	4	4	4	3	3	2	4	4	3	3	4
493	1	1	20	1	10	3	3	1	3	3	2	2	2	2	3	2	2	2	2	2	3	2	2	2	2	2	2	2	2	5	3	4	1	4
494	1	1	19	2	60	2	2	2	2	2	3	2	2	2	2	2	2	1	2	2	2	3	3	3	2	2	2	2	3	1	1	1	1	1
495	1	3	20	3	60	1	3	1	1	2	2	2	1	2	1	2	3	3	2	2	4	4	4	4	3	1	3	1	2	4	3	5	4	2
496	2	1	19	2	30	3	4	2	3	4	3	2	3	3	2	3	4	4	4	3	4	5	2	2	3	3	3	2	4	5	4	3	3	2
497	2	1	20	1	60	5	5	5	5	5	5	5	5	5	5	5	5	4	5	4	5	5	5	5	5	5	5	5	5	5	5	5	5	5
498	1	1	19	2	60	2	2	2	2	1	2	2	1	2	1	2	3	1	2	2	2	3	3	2	3	3	3	2	3	1	1	1	1	1
499	1	1	20	4	50	2	3	3	4	3	2	3	2	3	3	2	3	3	3	3	4	3	2	2	3	3	2	3	3	3	2	3	3	3
500	1	1	20	3	60	2	2	2	2	2	2	2	2	2	1	2	2	4	4	4	4	3	4	3	4	4	4	4	4	3	3	2	3	3

501	1	1	20	2	90	2	2	2	2	3	3	3	3	3	2	3	2	4	5	5	5	5	4	5	5	4	5	4	5	3	3	3	3	3
502	2	1	20	1	60	2	2	3	3	3	3	3	4	3	3	3	4	4	4	4	4	4	4	4	4	4	3	4	4	3	3	3	3	3
503	2	1	20	1	30	2	3	3	2	3	2	3	2	3	2	3	3	4	4	4	4	3	3	3	3	3	4	3	4	3	3	3	3	3
504	2	1	20	1	45	3	3	3	3	3	3	3	3	3	4	3	4	3	3	3	4	3	4	4	4	4	3	3	3	3	3	3	3	3
505	1	1	20	1	45	2	2	3	3	2	3	2	3	2	2	2	3	4	4	4	4	4	4	4	4	5	5	5	4	3	3	3	3	3
506	1	1	20	2	60	2	3	2	4	3	3	3	4	2	3	4	3	4	4	4	5	5	5	5	4	5	5	4	5	3	3	3	3	3
507	1	1	22	1	60	3	4	3	2	5	2	3	4	4	3	2	4	4	3	3	3	4	4	3	2	2	3	4	3	2	3	3	2	4
508	2	1	24	1	15	2	5	1	1	4	3	3	3	3	3	3	2	4	4	4	4	4	4	4	4	4	4	4	4	2	2	2	2	2
509	2	1	20	1	30	3	2	3	2	2	2	2	3	2	2	2	2	2	2	1	2	2	2	1	2	2	2	1	2	3	3	2	3	3
510	1	1	21	2	30	2	2	1	2	2	2	1	2	3	2	3	1	2	2	3	2	2	2	3	2	2	1	2	2	2	3	3	3	3
511	1	4	23	4	45	2	3	3	3	3	4	4	4	4	5	4	5	3	4	4	4	4	5	5	4	5	5	4	4	3	3	3	3	3
512	2	1	23	1	30	4	5	3	4	5	4	4	3	4	4	4	5	3	4	1	3	3	2	1	1	1	1	1	4	4	2	4	4	3
513	2	1	19	2	90	3	4	4	4	3	2	2	3	3	2	4	4	4	4	3	3	4	2	3	2	2	3	3	3	4	4	3	4	3
514	2	1	23	1	60	3	3	2	3	2	3	2	3	2	2	2	3	3	3	3	3	4	3	2	2	3	2	2	3	4	4	4	4	4
515	2	1	23	1	30	4	4	3	4	4	4	3	3	3	3	2	3	4	4	2	3	3	4	3	2	3	2	2	3	5	5	5	5	4
516	2	1	23	0	0	3	3	1	3	3	2	2	2	2	2	1	3	3	3	3	3	4	4	3	2	3	3	2	2	4	4	4	3	2
517	2	1	19	2	120	4	3	3	4	4	4	3	3	2	2	2	3	4	4	3	4	4	4	3	3	2	3	2	3	5	5	5	5	4
518	2	1	20	1	45	3	3	2	3	2	4	2	2	2	2	2	3	4	4	4	4	4	4	3	3	4	4	3	3	5	4	5	4	5
519	2	1	19	2	30	4	5	4	5	5	3	4	3	4	3	5	5	4	4	4	3	5	4	3	3	4	3	3	4	4	4	4	3	3
520	1	1	19	2	60	4	4	3	4	4	4	3	3	3	4	3	4	4	4	4	4	4	5	3	4	4	5	2	3	5	5	5	5	4
521	2	1	19	1	30	3	2	2	3	1	2	2	1	1	2	1	2	2	2	2	3	2	3	2	2	2	3	2	2	5	5	5	4	3
522	2	1	19	2	120	2	2	3	2	3	2	2	2	3	2	2	2	3	3	3	3	3	2	2	3	4	3	2	3	4	3	3	4	4
523	2	1	19	1	45	2	4	2	4	3	4	3	3	4	3	3	3	2	3	2	2	1	3	2	2	2	2	1	2	3	2	3	3	3
524	2	1	19	2	30	2	4	2	2	2	4	2	3	2	2	2	3	2	2	2	3	2	2	3	2	4	2	2	2	4	4	4	3	3
525	2	1	19	1	30	2	2	1	2	3	2	1	3	2	2	2	3	4	4	1	4	4	4	3	4	4	4	1	3	3	4	4	3	4
526	2	1	19	2	120	3	3	3	3	3	3	2	3	3	2	2	1	4	4	4	4	4	3	3	4	4	4	4	2	3	3	3	3	3
527	2	1	19	1	30	3	2	2	2	3	3	2	2	3	2	2	4	3	4	3	3	4	3	3	3	2	4	3	2	3	2	3	2	2
528	2	1	18	2	30	2	4	1	4	1	3	1	1	2	1	2	4	4	3	2	4	3	4	2	1	1	2	1	4	5	4	4	4	1
529	2	1	19	1	60	2	2	2	2	3	3	2	2	2	4	4	3	4	4	4	4	4	4	4	3	3	2	2	3	4	4	4	4	4
530	2	2	18	2	60	3	4	3	3	3	3	3	3	4	3	4	4	4	4	4	4	3	4	4	4	3	5	3	3	5	4	4	5	3
531	2	2	18	1	20	2	3	2	3	2	3	2	2	2	2	2	3	4	4	3	3	4	4	4	4	3	4	2	4	5	5	5	5	4
532	1	2	22	3	75	3	4	2	1	1	4	3	2	3	1	5	3	4	4	2	4	4	4	4	4	3	2	3	4	4	4	4	3	4
533	1	2	19	4	90	4	4	3	4	4	3	3	4	3	3	4	4	3	4	2	3	2	5	2	5	4	1	1	2	3	4	4	4	4
534	2	2	19	1	20	2	2	2	2	2	2	3	3	2	3	4	2	4	4	4	4	4	4	4	3	4	5	5	4	3	3	3	4	2
535	2	2	19	2	45	3	3	2	3	3	1	1	1	1	1	1	4	4	4	4	3	4	4	4	3	3	3	2	2	4	2	4	4	3
536	2	2	19	2	60	3	4	3	2	3	4	3	3	4	3	2	3	3	4	3	3	3	2	3	3	2	3	4	3	4	4	4	4	4
537	2	2	16	2	60	3	4	2	3	3	3	3	3	2	3	3	3	3	4	4	3	4	4	4	3	3	4	2	2	2	2	2	3	1
538	2	2	18	1	30	3	3	2	3	2	3	3	3	4	3	3	3	4	4	4	4	4	3	2	5	3	3	2	2	4	2	2	3	2
539	2	1	18	1	30	3	4	3	3	2	3	3	4	3	4	2	3	4	4	3	3	3	2	2	3	2	3	3	4	4	4	4	3	2
540	2	1	19	2	90	3	5	3	3	3	4	4	4	4	4	4	3	5	5	3	4	5	3	5	5	3	5	3	3	5	3	5	5	5
541	2	1	19	1	60	2	3	3	4	4	3	1	1	1	3	4	3	1	1	1	2	2	2	2	1	1	1	2	1	5	5	5	5	5
542	2	1	19	2	60	3	5	3	3	3	3	4	4	4	4	4	3	5	5	3	4	5	3	5	5	3	5	3	3	5	3	5	5	5
543	2	1	19	2	60	2	3	2	2	1	2	1	2	1	1	2	1	4	3	3	3	3	2	2	2	2	2	1	1	3	3	3	4	2
544	2	1	19	1	120	3	3	3	4	4	4	3	3	3	4	3	3	4	4	4	4	5	4	4	4	4	4	3	3	4	4	4	4	3
545	2	1	19	2	30	2	2	2	2	2	2	2	2	2	2	2	2	3	3	3	3	3	3	3	3	3	3	4	3	2	2	2	2	2
546	2	1	19	2	60	2	4	2	3	2	2	2	2	2	3	3	3	4	4	3	2	2	3	3	2	2	3	2	2	2	3	3	4	2
547	1	1	19	2	60	3	3	3	4	4	4	3	3	3	4	3	3	4	4	4	4	5	4	4	4	4	4	3	3	4	4	4	4	3
548	1	1	19	5	60	2	3	2	2	1	2	1	2	1	1	2	1	4	3	3	3	3	2	2	2	2	2	1	1	3	3	3	4	2
549	1	1	20	2	30	4	4	5	4	5	4	4	4	5	5	4	5	4	4	4	5	4	4	4	4	3	4	5	4	3	4	4	5	4
550	2	1	19	2	40	4	5	3	4	5	3	3	4	3	4	2	3	3	4	2	2	4	3	2	2	3	2	1	4	3	3	3	3	2
551	1	1	19	4	60	2	4	3	4	4	3	2	3	4	3	2	4	4	5	3	4	3	4	3	2	2	3	2	5	4	3	3	2	2
552	2	1	19	2	30	3	4	2	3	4	3	2	3	3	2	3	4	4	4	3	4	5	2	2	3	3	3	2	4	5	4	3	3	2
553	1	1	19	3	75	4	5	3	3	4	2	2	3	4	3	2	5	4	5	3	4	5	3	2	3	2	3	2	5	4	3	2	4	2
554	1	1	19	3	60	3	4	2	3	4	2	2	3	2	3	2	4	3	3	2	3	4	2	3	2	2	3	2	3	3	3	3	3	3
555	1	1	19	2	40	2	3	2	2	4	3	3	2	2	2	2	3	3	4	3	2	3	3	3	2	2	3	3	3	4	3	4	3	3
556	1	1	19	4	75	3	4	2	2	3	2	2	2	3	2	3	4	4	4	3	3	4	3	2	2	3	2	3	5	3	3	3	3	3
557	2	1	19	1	60	2	4	1	2	1	2	2	2	3	3	1	3	4	4	2	4	4	4	4	4	4	4	4	4	3	3	3	3	3
558	2	1	19	1	90	3	4	3	4	4	2	2	2	3	2	4	4	4	4	2	3	4	2	2	2	2	3	2	3	4	3	4	4	3
559	2	1	19	1	40	4	3	4	3	4	3	5	3	4	4	3	4	5	4	4	5	4	5	4	2	2	2	3	2	4	2	4	3	4
560	1	1	19	3	60	4	4	3	4	3	4	3	3	2	3	2	2	4	2	2	3	2	3	2	3	2	2	3	2	3	3	3	3	2
561	2	1	19	1	30	3	4	3	3	3	3	3	3	3	3	3	4	3	4	3	4	3	3	3	3	3	3	3	4	3	3	3	2	2
562	2	1	19	2	90	3	5	3	3	3	4	4	4	4	4	4	3	5	5	3	4	5	3	5	5	3	5	3	3	5	3	5	5	5
563	2	1	19	2	90	2	2	2	3	3	2	1	4	2	2	3	2	1	4	2	2	2	2	1	1	2	2	1	3	3	3	3	3	3
564	2	1	18	2	60	3	4	3	3	3	3	3	3	4	3	4	4	4	4	4	4	3	4	4	4	3	2	3	3	5	4	4	5	3
565	2	1	19	2	40	3	3	3	4	3	3	4	3	2	3	4	5	4	4	3	4	3	4	5	3	3	3	3	3	3	3	4	3	2
566	2	1	18	1	20	2	3	2	3	2	3	2	2	2	2	2	3	4	4	3	3	4	4	4	4	3	3	2	3	5	5	5	5	3
567	2	1	19	2	45	3	3	2	3	3	1	1	1	1	1	1	4	4	4	3	4	4	4	3	3	3	2	2	2	4	2	4	4	3
568	2	1	19	2	60	3	4	3	2	3	4	3	3	4	3	2	3	3	4	3	3	3	2	3	3	2	3	4	3	4	4	4	4	4
569	1	1	20	3	75	3	2	2	3	3	2	3	3	2	3	3	4	3	2	3	3	2	3	3	3	3	2	3	3	3	2	3	2	2
570	1	1	20	6	45	3	2	2	3	3	2	3	3	3	3	2	2	3	3	2	3	2	2	3	4	3	3	2	3	3	2	4	3	4
571	1	2	20	4	75	2	3	3	2	3	4	4	3	2	2	3	2	2	3	3	2	2	2	3	2	3	2	3	3	2	3	3	3	4
572	1	1	24	3	45	3	4	4	4	4	4	4	4	4	4	4	4	4	4	5	5	5	5	5	5	5	5	5	5	4	4	4	4	4
573	1	1	20	3	60	4	4	4	3	4	3	4	3	4	4	3	4	3	4	5	5	5	4	5	4	5	4	5	4	5	4	4	4	4
574	1	1	23	2	60	5	4	5	5	5	5	5	5	4	4	4	5	4	5	4	5	5	5	5	5	5	5	5	5	4	4	4	4	4
575	1	1	22	3	60	4	5	5	5	4	5	5	5	5	4	4	4	3	3	3	4	4	4	4	4	4	4	4	4	4	4	4	4	4
576	2	1	23	1	120	2	4	2	3	2	3	3	3	3	3	3	3	4	4	3	4	3	4	4	3	4	4	3	4	2	2	2	2	3
577	1	1	19	3	30	4	5	5	4	4	5	4	4	5	5	4	5	5	4	4	4	5	5	5	5	5	4	4	5	4	5	4	4	5
578	1	1	19	1	30	1	2	3	4	5	4	3	1	2	3	4	5	2	3	1	3	4	2	4	1	3	2	1	2	1	2	3	2	1
579	1	1	19	2	30	4	5	1	4	5	4	2	4	2	3	2	4	3	4	1	4	3	4	1	2	2	3	2	1	4	4	5	5	4
580	1	1	20	1	30	2	2	2	2	1	3	3	3	3	4	4	5	1	1	2	2	3	3	4	4	5	5	4	4	1	1	2	2	3
581	1	1	22	3	60	5	4	5	5	5	4	4	4	5	5	5	4	4	5	5	5	4	4	5	5	4	5	4	4	5	5	5	5	4
582	1	1	19	3	90	3	2	4	3	2	2	3	3	4	2	3	5	3	4	3	4	3	3	4	4	3	2	3	5	2	1	3	2	3
583	1	1	22	2	60	2	2	2	2	2	2	2	1	1	1	1	1	3	3	3	3	4	4	4	3	2	3	4	5	3	4	3	4	4
584	1	1	19	2	60	3	3	4	3	4	4	4	4	3	3	3	3	3	3	4	4	4	3	4	4	3	3	3	4	3	4	3	4	3
585	1	2	19	4	120	4	3	2	3	3	4	3	2	3	3	4	4	4	3	3	4	3	2	3	3	4	3	3	4	4	3	4	4	3
586	1	1	19	3	60	3	3	4	4	3	4	2	2	3	3	4	2	3	3	4	4	3	2	2	4	3	4	2	3	3	4	2	2	3
587	1	1	19	1	60	2	5	1	2	2	2	4	2	2	2	3	3	5	5	3	5	4	4	4	4	4	4	3	3	3	2	2	2	2
588	2	1	24	1	30	1	4	2	3	3	2	2	2	2	2	3	3	4	4	3	4	2	2	2	3	3	3	3	4	3	2	2	3	1
589	1	1	24	3	60	3	3	3	4	2	1	2	3	3	4	3	3	4	4	3	3	5	3	4	4	3	4	3	3	3	3	3	2	3
590	1	1	23	3	60	2	4	2	2	4	2	2	2	2	2	2	4	5	4	4	4	4	4	3	4	4	4	3	3	5	4	4	5	2
591	1	3	19	5	60	1	2	2	3	4	3	4	3	4	4	4	4	3	3	3	4	5	4	3	3	3	5	5	5	3	3	4	3	3
592	2	1	20	1	30	1	3	2	3	3	2	3	1	3	2	1	1	3	4	2	3	4	4	4	2	2	5	1	1	3	2	1	3	1
593	2	1	20	1	40	4	5	4	4	4	2	1	2	2	4	1	2	4	3	3	3	2	2	4	1	1	5	1	1	1	1	1	2	3
594	2	3	19	2	30	3	5	3	3	3	3	2	2	2	3	2	3	4	4	3	4	2	2	2	1	1	1	1	1	3	3	3	3	2
595	1	1	18	2	60	3	3	3	3	3	3	2	2	2	3	2	3	3	2	2	2	2	2	2	2	2	2	2	2	2	2	2	3	2
596	1	1	19	2	30	4	3	4	4	3	3	4	3	4	4	3	3	3	3	4	3	2	4	3	3	4	3	3	3	2	3	4	4	4
597	1	1	20	3	60	3	3	3	3	3	3	3	3	3	3	2	3	2	2	2	2	2	2	2	2	2	2	2	2	1	1	2	2	1
598	1	1	24	2	60	4	4	4	4	3	3	3	3	3	3	3	4	4	4	4	3	4	3	3	4	4	4	4	4	2	1	2	2	2
599	1	1	19	3	90	1	4	2	2	3	2	3	1	2	2	2	4	5	3	2	3	3	3	3	3	2	2	2	4	2	2	2	2	2
600	1	1	19	3	90	3	3	4	4	2	2	3	3	4	3	2	3	4	3	3	4	2	3	4	4	3	2	2	3	4	4	3	3	4
601	1	1	19	3	90	2	3	2	2	4	2	3	2	2	2	3	4	3	4	2	3	4	3	3	3	2	3	4	4	3	2	2	3	3
602	1	1	19	4	90	2	3	2	2	3	2	2	3	4	3	3	3	3	3	2	4	4	3	3	3	4	3	3	3	3	4	4	3	3
603	1	1	19	3	60	4	4	3	4	4	4	3	4	5	4	3	4	5	4	4	4	5	3	4	4	3	4	3	4	3	2	3	3	2
604	2	1	19	2	60	4	4	3	4	3	5	4	4	3	4	4	5	4	4	5	3	4	5	4	5	3	4	5	4	4	4	3	4	4
605	1	1	19	3	90	3	3	2	3	2	3	3	3	2	3	2	3	4	4	3	4	3	4	4	3	4	5	3	3	4	4	3	4	4
606	1	1	20	4	90	3	4	4	3	2	4	3	3	3	4	4	4	4	4	3	3	2	2	2	3	3	2	4	4	2	3	2	3	3
607	1	1	19	3	90	2	3	3	4	4	3	2	3	3	3	2	4	3	3	4	4	4	3	4	2	3	3	4	4	2	3	2	2	3
608	1	3	19	4	60	2	3	2	4	4	3	2	3	3	3	2	5	3	3	3	4	4	2	3	3	4	2	3	4	3	3	3	4	3
609	2	1	18	1	30	3	2	4	4	3	4	2	2	1	2	1	1	2	2	2	1	2	3	2	1	1	1	1	1	3	5	3	4	4
610	2	1	20	1	20	2	2	1	3	2	3	2	1	1	1	1	1	3	1	1	1	1	3	1	1	1	1	1	1	4	3	3	4	3
611	2	4	24	1	30	1	5	1	3	1	1	1	1	1	1	2	4	5	5	5	5	5	5	5	5	5	5	5	5	4	3	4	3	3
612	2	1	20	1	20	1	2	1	1	2	1	1	1	1	1	1	2	3	3	4	3	3	3	4	3	4	3	3	4	3	2	1	3	1
613	2	2	21	1	30	3	4	3	3	2	2	3	4	2	4	2	2	3	4	3	4	3	2	4	4	3	4	2	3	4	4	4	4	2
614	2	1	21	1	20	2	1	1	2	2	2	1	1	1	1	1	1	2	1	1	2	2	1	1	2	1	1	1	1	3	3	3	3	2
615	2	1	21	1	210	4	5	4	4	4	4	4	4	4	4	4	3	3	3	2	3	2	3	2	3	2	3	3	2	4	3	4	4	3
616	2	1	19	1	30	2	2	2	2	2	2	2	2	2	2	2	2	4	3	3	4	3	3	3	3	3	3	3	3	3	2	4	2	1
617	2	1	22	0	0	2	2	2	2	2	2	2	2	2	2	2	2	3	3	3	3	2	2	2	2	2	2	2	3	3	3	4	4	4
618	1	1	23	2	20	3	3	3	3	3	3	3	3	3	3	3	3	4	4	3	4	3	4	3	4	3	4	4	4	5	4	3	5	4
619	1	1	20	4	60	3	3	3	3	3	3	3	3	3	3	3	3	4	4	3	4	4	4	3	3	3	3	3	4	4	4	4	4	4
620	1	1	20	5	60	2	2	2	2	2	2	2	2	2	2	2	2	4	4	4	4	4	4	4	4	4	4	4	4	4	4	4	4	4
621	1	1	21	0	0	2	2	2	2	2	2	2	2	2	2	2	2	4	4	4	4	4	4	4	4	4	4	4	4	3	3	3	3	3
622	1	1	22	2	60	1	1	1	1	1	1	1	1	1	1	1	1	4	4	4	4	3	4	4	4	4	4	4	4	4	4	4	4	4
623	1	1	23	2	60	2	2	2	2	2	2	2	2	2	2	2	2	5	5	5	4	2	3	3	4	4	4	4	4	4	4	4	4	4
624	1	1	21	2	60	2	2	2	2	2	2	2	2	2	2	2	2	4	4	4	4	4	4	4	4	4	4	4	4	4	4	4	4	4
625	1	1	21	4	60	4	4	4	4	4	3	4	4	4	4	4	3	5	5	5	5	4	4	4	4	4	4	4	4	3	4	4	3	3
626	1	1	21	2	60	4	3	4	3	3	3	4	4	4	4	4	4	4	4	4	5	4	4	5	5	5	5	5	5	4	3	3	3	3
627	1	1	23	5	40	1	1	1	1	2	2	2	2	2	2	2	2	5	4	5	5	5	5	5	5	5	4	1	2	4	4	4	4	3
628	2	1	21	1	30	3	5	2	3	3	5	3	3	2	5	3	4	4	4	2	3	4	3	4	3	2	4	2	4	5	3	4	3	4
629	2	1	20	1	30	3	3	3	2	3	2	3	3	2	1	3	2	4	4	3	3	3	4	4	4	4	3	4	3	5	4	5	5	4
630	2	1	20	1	30	3	3	3	4	3	2	3	2	3	2	3	3	3	4	2	3	2	3	3	3	2	3	3	3	2	3	3	3	2
631	2	1	20	1	15	2	2	2	2	2	2	2	2	2	3	3	3	3	3	3	3	3	3	3	3	3	3	3	4	5	5	5	5	5
632	2	1	20	1	30	3	3	3	3	4	3	3	4	4	4	3	3	3	4	4	4	3	3	4	4	3	3	3	4	5	5	5	5	5
633	2	2	21	1	30	1	3	3	3	3	3	3	3	3	3	3	3	4	4	3	3	3	3	3	4	4	4	4	4	2	2	2	2	2
634	1	1	18	3	60	3	1	1	2	1	1	1	1	1	1	1	3	5	2	5	5	5	5	5	5	2	5	1	1	5	1	5	3	1
635	1	2	18	1	60	2	2	3	3	1	2	3	3	3	2	3	3	4	3	4	4	1	3	3	3	3	3	4	4	5	4	5	5	5
636	2	1	18	1	30	3	4	2	3	2	3	3	3	2	3	3	3	3	2	3	4	3	2	3	2	3	2	3	2	2	3	3	3	2
637	2	1	19	2	90	3	5	3	3	3	3	4	4	4	4	4	3	5	5	3	4	5	3	5	5	3	5	3	3	5	3	5	5	5
638	1	1	22	3	90	5	5	5	5	4	5	5	4	5	4	4	4	5	5	4	4	4	4	4	5	4	4	4	5	5	5	5	5	5
639	2	1	19	2	60	5	5	2	4	4	3	4	3	3	4	2	3	4	3	3	5	4	3	4	3	3	4	5	5	3	4	2	4	5
640	2	1	19	1	180	3	4	2	3	3	3	3	3	3	3	3	3	3	3	3	3	3	3	3	3	3	3	3	3	4	2	3	3	3
641	2	1	19	2	120	3	4	2	3	2	3	3	4	4	3	2	4	3	4	3	4	4	4	3	4	3	3	3	4	4	3	4	2	3
642	2	3	19	2	90	2	3	2	3	3	3	3	3	3	2	2	2	1	2	2	2	2	2	2	1	2	2	1	2	3	2	3	3	3
643	1	2	20	2	60	1	3	1	2	4	3	1	1	1	1	3	3	5	5	5	5	4	4	4	5	5	4	4	4	5	5	5	5	5
644	1	4	20	1	30	5	4	4	4	1	1	3	1	1	3	3	3	3	4	3	3	4	1	2	2	3	3	3	3	4	4	3	4	4
645	2	1	19	2	120	2	3	2	3	3	2	2	2	2	3	2	3	3	3	2	3	2	3	3	3	3	4	3	2	3	2	2	1	3
646	2	1	21	1	150	1	4	1	3	2	2	1	1	1	1	1	1	5	5	3	3	2	4	3	2	1	3	1	1	3	2	2	2	2
647	1	1	21	2	120	2	3	2	3	4	1	2	2	1	2	3	3	4	4	4	4	5	3	3	3	3	3	3	5	5	5	5	5	4
648	2	2	21	1	60	5	4	5	4	5	5	5	3	3	3	4	4	5	5	5	4	4	3	3	4	4	5	5	5	5	5	5	5	3
649	1	2	20	2	90	4	5	3	3	3	2	3	3	2	3	3	2	4	5	4	4	5	4	4	4	5	4	5	5	3	4	4	5	4
650	2	2	25	1	60	2	4	3	3	3	3	3	3	3	3	3	3	4	4	4	4	4	3	3	4	4	3	4	3	3	3	3	3	3
651	2	2	21	7	30	2	2	3	2	2	1	2	3	2	1	2	3	4	3	4	4	4	3	4	4	3	3	3	4	4	4	5	4	2
652	2	2	24	1	30	1	3	1	3	1	2	2	2	2	2	3	3	3	4	3	3	4	2	3	3	3	3	3	3	3	2	3	4	2
653	2	2	21	1	60	2	4	1	3	2	4	4	2	3	2	2	4	3	4	4	3	4	4	3	5	2	3	4	4	4	2	5	4	1
654	2	1	21	1	20	1	2	1	2	2	2	1	2	2	2	1	1	1	2	1	1	1	2	2	2	2	1	1	2	4	4	3	2	1
655	1	1	22	2	90	1	2	2	1	1	2	1	1	2	2	2	3	3	3	2	4	3	3	2	2	3	3	2	2	3	3	2	2	2
656	2	1	19	2	120	4	4	4	5	4	5	4	4	4	4	3	3	3	4	3	3	3	4	4	4	3	4	2	3	4	3	4	4	3
657	1	1	23	3	60	2	1	2	3	4	2	3	2	2	2	2	3	3	3	2	4	4	3	3	3	4	4	2	4	2	1	1	1	2
658	1	1	20	4	120	4	3	4	3	3	3	4	5	3	4	3	3	4	4	3	3	5	3	4	3	3	4	3	3	3	3	3	3	3
659	1	1	19	5	120	2	4	3	4	3	3	3	2	4	2	3	3	3	4	3	2	3	4	4	4	3	4	3	4	5	4	5	5	3
660	2	1	19	1	30	3	4	3	4	5	3	3	2	3	4	4	4	3	2	2	2	4	4	3	2	4	3	2	3	3	3	2	4	3
661	1	1	24	2	30	2	2	2	2	2	2	2	2	2	2	2	2	2	2	2	2	2	2	2	2	2	2	2	2	5	5	5	5	5
662	2	2	19	2	90	4	4	4	4	4	4	4	4	4	3	3	3	4	4	4	4	4	4	4	4	4	4	4	3	4	4	4	4	4
663	2	1	19	2	90	3	3	3	3	3	3	3	3	3	3	3	3	4	4	3	4	4	4	4	3	3	3	3	3	4	3	3	4	2
664	2	1	20	1	60	4	4	4	4	4	4	4	2	3	3	2	2	2	2	2	3	2	2	2	2	2	2	2	2	2	2	2	2	2
665	1	1	20	1	40	2	2	1	1	1	1	1	1	1	1	1	1	2	2	2	2	2	2	2	2	2	2	2	2	3	3	3	3	3
666	2	1	20	1	40	2	2	2	2	3	3	3	3	2	2	2	2	2	2	2	2	2	2	2	2	2	2	2	2	3	3	3	3	2
667	2	1	20	1	60	3	3	3	3	2	2	2	3	3	3	2	2	3	3	3	3	2	2	2	3	2	2	2	2	4	4	4	3	2

668	2	1	20	1	45	1	1	1	1	1	1	1	1	1	1	1	1	2	2	2	2	1	2	2	2	2	2	2	2	2	2	2	2	2
669	1	1	21	3	#NULL!	4	5	3	3	4	3	3	4	4	3	4	3	3	3	3	3	3	3	3	3	3	3	3	3	3	3	4	4	3
670	1	2	22	3	#NULL!	4	5	5	5	5	3	3	4	4	5	4	4	4	2	3	4	4	4	4	4	4	4	4	4	4	1	2	2	1
671	1	3	22	1	#NULL!	2	3	2	2	4	3	3	3	2	3	2	3	2	3	2	2	3	3	3	3	2	3	2	3	4	4	4	4	4
672	1	1	21	1	#NULL!	2	2	1	2	1	1	1	1	1	1	1	1	5	5	5	5	3	4	5	3	2	4	1	3	3	1	2	5	1
673	1	2	23	5	#NULL!	2	4	2	2	2	3	2	1	1	1	3	3	3	5	3	3	4	3	4	1	2	1	4	4	2	1	1	2	1
674	2	1	22	1	#NULL!	2	4	3	4	2	3	3	2	2	2	2	3	2	2	2	2	2	3	2	2	2	2	2	2	2	2	2	2	3
675	1	1	24	1	60	1	2	1	3	1	5	5	3	3	3	5	5	3	3	3	3	4	2	3	3	2	3	4	3	5	5	5	5	5
676	1	2	22	2	20	2	3	2	2	2	3	3	3	2	3	3	3	2	3	2	2	2	3	3	3	2	2	2	4	3	3	2	2	3
677	1	1	25	1	60	1	2	2	2	2	2	2	2	2	1	1	4	3	3	4	3	3	3	3	3	4	4	3	5	3	3	3	3	3
678	2	1	24	1	60	2	2	3	3	3	2	3	2	3	3	2	2	3	3	3	2	3	3	3	4	4	4	3	3	3	4	4	4	3
679	2	1	20	0	0	2	3	2	2	3	3	3	3	2	2	2	2	3	3	2	3	3	2	2	2	3	3	3	3	3	3	3	2	2
680	1	1	23	3	30	3	3	2	2	2	3	3	2	2	2	3	3	3	3	3	3	3	2	2	2	2	3	2	3	4	3	3	3	3
681	1	1	20	7	120	2	2	2	2	2	2	2	2	2	2	2	2	5	5	5	5	5	4	4	4	4	4	1	1	5	2	3	5	3
682	1	1	20	7	120	5	5	5	5	5	5	5	5	5	5	5	5	5	5	5	5	5	5	5	5	5	5	1	1	5	4	4	5	5
683	1	1	20	7	120	5	5	5	5	5	5	4	4	4	4	3	3	5	5	5	5	5	5	4	4	4	4	2	2	5	5	5	5	5
684	1	1	24	7	120	4	4	4	4	4	4	4	4	4	4	4	3	5	5	5	5	5	5	5	5	5	5	1	1	5	5	5	5	5
685	1	1	21	2	120	2	2	1	2	2	1	2	2	1	2	1	2	4	4	2	5	5	4	2	4	1	4	1	2	4	2	3	4	4
686	2	1	19	2	120	2	2	2	2	2	2	2	2	2	2	2	2	5	5	5	5	5	5	4	4	4	4	2	3	4	4	4	4	4
687	2	1	19	2	120	4	4	4	4	4	3	3	3	3	3	3	4	4	4	4	4	4	4	4	4	4	4	3	3	5	2	2	5	4
688	2	1	23	1	90	4	4	2	4	4	3	3	3	3	3	3	3	4	4	2	4	4	3	1	1	3	1	1	3	5	2	2	5	5
689	1	1	21	3	60	3	3	3	2	1	3	2	3	2	3	2	2	4	4	3	5	4	4	4	4	4	4	4	4	4	4	3	2	2
690	2	1	19	2	45	4	5	4	3	4	4	3	3	3	4	4	3	4	4	5	3	4	3	3	3	4	4	3	5	4	3	3	4	3
691	2	1	19	2	90	4	3	2	3	2	2	2	2	2	2	3	2	4	3	3	3	3	3	3	2	2	3	2	2	4	4	4	4	3
692	2	1	19	1	30	3	3	2	3	1	3	1	1	1	1	1	1	3	3	2	2	2	3	1	1	1	1	1	1	4	4	4	4	4
693	1	1	20	2	40	3	2	3	3	3	2	2	2	3	2	2	3	4	4	4	3	3	3	3	4	3	3	3	4	4	4	4	4	2
694	2	1	20	1	30	3	4	3	4	4	4	3	3	3	3	3	4	4	4	3	4	4	3	4	4	3	4	3	4	5	5	5	5	3
695	1	1	20	1	60	4	4	3	4	4	3	3	4	3	3	3	3	4	4	4	4	4	4	3	4	4	4	4	4	4	4	4	5	3
696	1	1	20	4	120	4	4	3	3	4	3	3	3	3	3	3	4	5	5	3	4	5	4	4	4	5	4	3	4	5	5	5	5	3
697	1	1	21	5	160	3	3	3	3	4	3	3	3	3	3	3	4	4	4	4	4	4	4	4	4	4	4	3	4	5	5	5	5	4
698	1	1	22	5	120	3	4	3	3	4	3	3	3	3	3	3	5	5	5	4	4	5	4	4	4	4	4	4	5	5	5	5	4	4
699	1	1	20	2	120	2	3	2	2	3	2	2	2	2	2	3	4	4	4	4	4	3	3	3	3	3	3	3	4	4	3	4	3	3
700	1	1	20	1	30	2	1	2	2	1	1	1	2	2	1	2	1	4	5	5	5	5	4	2	1	1	1	1	1	4	5	5	4	4
701	2	1	20	1	40	4	3	4	4	4	3	4	5	4	5	4	5	5	5	5	5	5	4	4	5	4	5	4	5	5	5	5	5	5
702	1	1	21	2	60	2	1	2	2	1	1	1	2	2	1	2	1	4	5	5	5	5	4	2	1	1	1	1	1	4	5	5	4	4
703	1	1	23	2	30	1	3	3	2	1	2	2	2	2	2	2	2	4	4	4	4	4	4	4	4	4	4	4	4	4	4	4	4	4
704	1	1	22	3	60	4	5	3	5	3	2	2	3	2	3	4	5	5	5	4	5	4	4	4	3	3	3	4	5	4	3	3	5	2
705	1	1	23	3	30	4	5	3	5	3	2	2	3	2	3	4	5	5	5	4	5	4	4	4	3	3	3	4	5	4	3	3	5	2
706	1	2	21	2	60	3	4	5	3	4	3	2	2	1	1	4	5	4	3	4	3	4	3	4	4	3	4	4	2	5	3	5	3	3
707	1	1	23	2	120	2	4	2	2	3	2	2	2	2	2	2	4	4	4	4	4	4	4	4	4	4	4	3	3	4	3	3	4	3
708	1	1	23	2	40	3	3	2	2	3	3	2	2	2	2	3	3	2	3	3	3	2	2	3	3	2	3	2	3	4	4	3	4	3
709	1	4	24	7	30	3	3	4	3	4	3	3	2	3	3	4	4	3	3	2	3	3	3	3	3	3	3	3	3	3	3	3	4	3
710	1	1	25	1	180	3	4	4	4	4	4	4	4	4	4	4	4	5	5	5	5	5	5	5	5	5	5	5	5	4	4	5	4	3
711	1	1	24	2	120	2	4	3	3	4	3	3	3	3	3	2	4	4	5	4	4	5	5	5	4	4	4	4	3	4	4	4	4	4
712	2	1	24	1	60	2	3	3	2	2	3	2	3	2	2	2	3	3	2	3	3	3	3	2	3	3	3	2	3	3	3	3	3	3
713	1	1	20	3	120	3	4	3	3	4	3	3	3	3	3	3	3	4	4	3	4	3	4	4	3	3	4	4	3	4	3	4	3	3
714	2	1	20	1	60	2	2	2	1	1	1	1	1	2	2	1	2	2	3	3	3	2	3	2	2	3	3	3	3	4	4	4	4	3
715	1	1	23	1	120	2	3	3	3	3	3	2	2	3	2	2	3	4	3	3	4	3	4	3	3	4	3	3	3	4	4	3	4	3
716	1	1	20	2	120	2	2	2	3	3	3	3	2	3	3	3	3	4	3	4	3	3	3	3	3	3	3	4	4	4	4	3	4	4
717	1	1	20	2	30	2	2	2	2	2	2	2	2	2	2	2	2	3	3	4	4	2	3	3	3	3	3	3	2	4	3	3	3	2
718	1	1	23	7	60	2	2	2	2	2	2	2	2	2	2	2	3	4	4	4	4	3	4	4	4	4	4	3	3	4	4	4	4	4
719	1	1	21	2	120	3	3	3	3	2	3	3	3	3	3	3	3	4	4	4	3	4	4	4	4	4	4	4	4	4	4	4	4	3
720	1	1	20	5	60	2	2	2	2	2	2	2	2	2	2	2	3	3	3	3	3	2	2	2	2	3	3	3	3	4	4	4	3	3
721	2	1	20	1	30	2	2	2	3	2	2	3	2	2	3	3	3	3	3	3	4	3	3	3	3	3	3	4	4	4	4	4	3	3
722	1	2	21	2	60	3	4	5	3	4	3	2	2	1	1	4	5	4	3	4	3	4	3	4	4	3	4	4	2	5	3	5	3	3
723	1	1	23	1	30	1	4	1	2	2	1	1	2	1	1	1	1	2	2	2	2	2	2	2	2	2	2	2	2	4	3	4	4	3
724	1	1	23	1	30	4	4	4	4	4	4	3	4	4	4	5	5	4	4	4	4	4	5	5	5	5	5	4	4	3	3	4	4	4
725	1	1	21	1	120	2	4	3	4	4	3	3	4	3	4	1	3	3	3	2	3	4	1	2	2	1	2	1	1	4	1	3	4	3
726	1	3	23	1	40	3	2	1	1	3	1	1	1	1	1	1	1	4	2	4	3	4	3	4	3	4	3	4	3	3	1	4	4	1
727	1	1	21	2	60	2	3	2	3	2	3	4	3	3	3	2	4	4	4	3	4	2	3	3	4	4	4	4	3	4	4	4	4	4
728	1	1	22	6	90	4	4	2	3	4	4	4	3	3	3	3	2	4	4	4	4	4	4	4	4	4	4	4	3	3	3	3	4	3
729	1	2	23	1	28	2	3	1	3	3	1	2	3	1	1	1	1	3	5	3	4	4	4	4	5	3	5	2	1	4	4	5	5	4
730	1	2	23	7	#NULL!	1	1	1	1	1	1	1	1	1	1	1	1	1	1	1	1	1	1	1	1	1	1	1	1	3	3	3	4	5
731	1	1	20	6	40	2	1	2	2	1	1	2	1	2	2	2	2	5	4	5	5	4	5	5	4	3	4	3	3	5	5	5	5	5
732	1	2	20	5	60	4	3	3	3	4	4	5	3	3	4	3	4	5	4	4	5	5	4	3	4	5	4	3	3	5	5	5	5	5
733	1	1	19	5	90	3	3	4	3	3	4	2	3	2	2	3	4	4	4	3	4	3	3	2	3	3	4	3	4	3	2	2	4	3
734	1	1	20	6	60	2	4	1	2	2	1	1	1	1	2	2	2	4	3	3	4	4	5	5	4	4	3	4	4	5	4	4	3	4
735	1	1	19	5	60	4	5	4	4	4	5	4	4	5	4	4	5	4	5	5	5	4	4	4	3	4	4	5	4	4	5	4	3	4
736	1	1	24	2	15	3	3	3	2	2	2	3	3	3	3	2	2	3	2	3	3	4	3	2	3	3	3	2	2	3	2	3	2	3
737	1	1	19	5	60	2	3	2	3	2	2	3	2	2	2	2	2	3	3	3	3	3	3	3	3	3	3	2	3	2	2	1	2	1
738	1	1	20	4	60	2	3	2	3	2	4	1	1	2	2	1	2	4	4	3	5	5	4	3	3	4	5	2	5	1	1	1	2	1
739	1	1	21	3	45	1	1	1	1	2	2	2	2	2	2	1	1	3	4	2	2	3	3	3	3	3	2	2	1	3	3	2	2	2
740	1	3	24	3	90	2	3	3	2	3	2	2	2	3	2	2	2	3	2	2	3	2	2	3	3	4	3	3	3	3	3	4	3	4
741	1	1	24	1	60	2	1	2	2	2	2	1	3	3	2	3	2	2	3	2	1	3	2	3	2	1	2	3	2	2	1	2	2	1
742	1	1	20	4	60	1	3	2	3	4	3	3	4	2	2	3	5	2	4	1	3	3	3	3	4	3	4	2	3	3	3	3	4	1
743	1	1	19	3	90	2	4	2	3	4	3	1	1	1	1	1	1	3	3	2	3	2	3	1	1	1	1	1	1	4	4	4	4	2
744	1	1	19	3	60	3	3	2	3	3	2	2	1	2	1	2	1	4	2	3	4	2	2	2	1	1	1	1	1	3	2	3	3	1
745	1	1	19	2	160	2	2	2	3	4	2	1	1	1	1	1	1	4	2	3	4	3	1	1	1	1	1	1	1	4	4	4	4	1
746	1	1	19	7	30	1	1	1	2	1	1	1	1	1	1	1	1	2	1	1	2	1	1	1	1	1	1	1	1	4	3	3	3	1
747	1	1	19	3	120	2	1	1	2	2	1	1	1	1	1	1	1	3	2	2	3	2	3	1	1	1	1	1	1	4	3	4	4	1
748	1	1	22	7	15	1	1	1	2	2	2	2	1	1	1	1	1	2	1	1	2	2	2	2	1	1	1	1	1	4	3	4	4	1
749	1	1	22	2	60	2	1	1	2	2	2	1	1	1	1	1	1	3	2	1	3	2	3	1	1	1	1	1	1	4	4	4	4	2
750	2	1	19	2	90	4	4	4	4	4	4	4	1	3	1	2	2	4	4	3	3	3	3	1	1	1	1	1	3	4	4	4	4	1
751	1	1	19	4	180	3	4	1	2	4	2	2	1	2	1	1	1	4	4	4	4	4	4	4	1	1	1	1	1	4	3	3	3	1
752	1	1	24	3	20	2	3	2	3	2	4	1	1	2	2	1	2	4	4	3	5	5	4	3	3	4	5	2	5	5	2	5	3	3
753	1	1	24	0	0	1	1	1	1	2	2	2	2	2	2	1	1	3	4	2	2	3	3	3	3	3	2	2	1	2	1	1	2	3
754	1	1	19	3	90	1	2	1	1	2	2	2	1	1	1	1	1	3	3	3	3	2	2	2	3	3	2	2	1	1	1	1	2	1
755	1	1	19	3	60	2	2	3	2	1	3	2	1	2	2	2	1	2	3	2	3	3	2	3	1	2	2	2	2	3	3	2	2	2
756	1	1	19	4	60	2	3	2	4	3	4	3	4	3	4	4	3	5	4	4	4	4	4	4	4	4	4	4	4	3	3	3	3	3
757	1	1	19	6	90	2	5	3	2	3	2	2	2	2	2	2	4	5	4	3	4	4	4	3	3	2	2	1	3	4	1	3	4	2
758	1	1	19	1	150	2	1	1	1	2	1	2	1	1	1	2	1	5	5	4	4	4	3	2	2	1	4	1	1	3	2	2	5	1
759	2	1	19	1	30	1	1	1	1	2	3	1	1	3	3	3	1	1	1	1	1	1	3	1	3	1	1	1	1	5	2	5	4	2
760	1	1	23	5	30	3	4	3	4	4	4	4	4	4	4	4	3	5	5	5	5	4	4	5	5	5	4	4	2	3	1	2	4	2
761	1	4	21	5	70	2	4	1	2	3	3	3	3	3	3	3	3	4	4	3	4	4	4	4	4	3	3	4	3	2	1	2	3	2
762	1	1	21	6	120	2	3	1	1	2	2	2	2	2	2	1	3	3	3	2	3	4	2	2	3	2	3	2	4	4	3	4	4	1
763	1	4	24	4	30	3	4	3	4	4	3	4	3	4	4	4	4	3	4	3	3	4	3	3	3	3	3	2	2	3	3	3	3	3
764	1	1	21	5	90	3	4	3	4	4	4	4	4	4	4	4	3	5	5	5	5	4	4	5	5	5	4	4	2	3	1	2	4	2
765	1	1	21	3	60	1	3	2	3	3	4	3	3	4	4	2	3	5	5	4	4	4	5	4	3	4	4	3	4	5	4	5	5	4
766	1	1	21	5	45	1	2	2	2	2	2	2	2	2	2	2	2	4	4	4	4	4	4	4	4	4	4	4	4	3	4	4	4	3
767	1	1	19	2	30	2	2	2	1	2	2	2	1	3	2	2	2	3	4	3	4	3	4	4	4	4	4	3	3	2	3	4	4	3
768	1	4	20	5	30	2	2	2	2	2	4	4	4	4	4	4	3	2	2	2	2	2	2	2	2	2	2	2	2	3	3	3	3	3
769	2	1	20	1	30	3	5	3	3	5	3	3	5	3	5	5	5	5	5	5	5	5	5	5	5	5	5	5	5	3	3	3	1	1
770	2	1	19	2	45	4	4	4	4	4	3	4	3	4	3	3	3	4	4	3	4	3	3	3	3	3	3	3	3	4	4	4	4	4
771	2	1	19	1	30	3	4	3	3	3	3	3	3	3	3	3	4	3	4	4	4	4	4	4	4	4	4	4	4	3	2	3	2	3
772	1	1	23	4	60	3	5	3	4	4	4	3	2	2	3	3	4	4	4	2	4	3	4	2	2	3	3	2	4	4	3	3	3	3
773	1	4	19	2	60	1	1	1	1	1	2	1	1	1	1	2	2	5	4	3	4	4	3	3	3	3	3	2	2	2	4	3	4	3
774	2	1	19	2	45	4	5	4	5	4	4	4	5	3	3	2	1	4	4	5	5	5	4	3	4	3	3	4	2	4	3	3	2	2
775	2	1	19	2	30	4	5	5	5	5	5	4	3	4	5	3	2	3	3	3	4	3	4	3	2	2	3	3	3	4	2	3	2	3
776	1	1	23	3	90	4	5	4	3	4	4	3	3	3	4	5	4	4	4	5	4	5	4	3	3	5	4	4	4	4	3	3	4	3
777	1	2	20	3	180	3	4	2	3	5	5	4	5	5	3	5	4	3	4	4	5	3	5	5	3	4	2	3	5	4	5	4	5	5
778	2	1	21	1	60	2	2	2	2	2	2	2	2	2	2	2	2	3	3	2	3	2	2	2	3	3	2	2	3	3	3	3	3	3
779	1	1	23	5	60	4	3	4	4	3	5	3	4	5	4	5	4	3	4	3	3	4	5	3	4	4	4	5	4	4	3	3	4	3
780	1	2	22	2	20	2	3	5	4	3	5	3	4	4	3	5	4	3	3	4	3	5	5	3	4	3	4	3	3	2	3	5	5	5
781	1	1	22	2	90	4	5	4	4	4	4	4	4	4	4	4	4	4	4	4	4	4	4	5	4	3	4	5	4	4	4	4	3	3
782	1	1	20	3	35	3	4	3	3	3	3	3	3	3	3	3	5	4	4	4	4	4	4	5	4	4	4	4	4	5	5	4	4	2
783	1	2	22	2	60	1	3	1	1	2	1	2	1	1	1	1	1	3	4	3	3	3	1	3	3	3	3	1	1	5	2	5	5	1
784	1	2	23	4	40	1	1	1	2	2	1	2	1	1	1	1	2	3	2	2	2	3	2	2	1	2	1	1	2	2	2	2	3	2
785	1	1	20	7	120	3	5	3	4	4	3	2	4	4	3	3	3	4	4	4	4	4	4	4	4	4	4	4	4	4	4	4	4	4
786	1	1	21	5	60	1	1	1	1	1	1	1	1	1	1	2	2	4	4	4	4	5	5	5	4	4	4	4	3	4	4	4	4	3
787	1	1	21	3	60	2	3	2	2	3	3	3	3	3	3	3	3	2	2	3	2	3	3	2	3	3	2	3	3	4	4	4	4	4
788	2	1	19	1	15	3	2	2	3	2	2	2	2	2	2	2	3	3	3	2	3	3	4	3	3	3	3	3	3	5	4	4	5	4
789	2	2	19	1	10	3	4	2	2	2	3	2	2	3	3	3	4	3	4	3	4	3	4	4	4	4	4	3	5	5	4	4	5	3
790	1	1	20	4	60	3	3	3	3	3	3	3	3	3	3	3	3	4	3	3	4	3	3	3	3	3	4	3	4	4	4	4	4	3
791	1	1	20	7	120	5	4	5	4	5	4	5	4	5	4	5	4	5	4	5	4	5	4	5	4	5	4	5	4	3	4	5	4	3
792	1	1	21	6	60	3	3	3	3	4	3	2	3	2	4	3	2	3	2	2	3	4	3	2	4	3	2	3	3	2	3	2	3	2
793	1	1	20	4	30	4	4	3	3	4	4	4	3	4	4	4	4	4	4	5	4	4	4	4	3	4	4	4	4	4	4	5	4	4
794	1	1	21	3	45	2	3	4	3	4	5	3	3	2	3	4	4	4	4	5	3	3	2	2	3	3	4	3	4	3	4	4	5	5
795	1	1	20	5	10	1	1	1	3	4	1	2	1	1	1	1	5	3	3	2	5	5	5	4	2	2	1	1	5	5	4	5	5	4
796	1	1	21	3	30	3	3	3	3	2	2	3	3	3	2	2	2	3	3	2	3	3	3	3	2	3	3	3	3	3	3	2	3	2
797	1	1	20	5	30	3	3	3	3	3	2	3	2	2	3	2	2	3	3	3	3	2	3	3	3	2	3	3	3	3	3	3	3	3
798	1	1	22	5	45	2	2	3	2	2	3	3	3	2	3	3	3	2	2	2	3	3	2	2	2	2	3	3	3	3	3	3	3	3
799	1	3	20	5	30	2	3	3	2	3	3	2	3	3	3	3	3	3	3	3	3	3	3	2	3	2	2	3	2	3	3	3	3	3
800	1	1	20	5	40	2	2	3	2	3	3	2	2	3	3	3	2	2	2	3	3	3	2	2	3	3	3	3	3	3	2	2	2	2
801	1	1	20	2	45	1	3	2	2	3	2	2	3	2	3	3	3	3	3	3	2	2	2	3	3	2	3	3	2	3	2	2	3	3
802	2	1	22	1	10	2	4	1	4	3	3	3	3	3	3	3	4	1	2	2	2	2	2	2	2	2	2	2	2	5	2	3	5	5
803	1	1	20	2	60	2	1	3	2	2	2	1	2	2	1	2	3	2	3	3	4	5	4	3	4	3	2	3	2	1	2	3	2	2
804	1	1	21	3	30	3	2	4	2	4	3	3	2	4	3	2	4	3	3	2	4	4	2	3	3	2	4	2	3	3	3	2	2	4
805	1	1	20	7	120	5	5	5	4	5	4	5	4	5	4	5	4	4	5	4	5	4	5	4	5	4	5	4	5	4	5	4	5	4
806	1	3	24	7	180	1	1	1	1	1	1	1	1	1	1	1	1	1	1	1	1	1	1	1	1	1	1	1	1	1	1	1	1	1
807	1	1	20	3	90	3	3	3	3	3	3	3	3	3	3	3	3	3	2	3	3	3	3	3	3	3	3	3	3	3	3	3	3	3
808	1	1	19	4	50	4	4	3	4	3	3	4	3	4	4	3	4	5	4	3	4	4	3	4	3	4	4	3	4	4	3	3	3	3
809	1	1	19	4	60	4	5	3	4	4	3	3	4	4	3	3	4	5	4	3	4	4	5	4	5	4	5	3	4	5	3	5	5	5
810	1	1	19	5	25	3	3	3	3	3	3	3	3	4	3	4	3	4	3	4	5	4	4	3	5	4	3	4	5	4	5	3	4	4
811	1	1	19	3	60	4	4	2	3	4	4	3	4	3	4	3	2	4	4	2	4	4	4	4	4	4	4	2	5	4	4	4	4	4
812	1	1	19	3	60	3	3	3	3	4	4	4	3	4	4	4	3	4	5	5	5	4	4	4	3	3	4	5	5	4	3	4	3	3
813	1	1	19	4	30	4	4	4	4	4	4	4	4	4	4	4	4	4	4	4	4	4	4	4	4	4	4	4	4	4	4	4	4	4
814	1	1	19	4	60	2	3	1	3	4	2	3	3	3	3	2	4	4	4	3	3	3	2	4	4	3	3	3	3	3	3	4	4	4
815	1	1	19	3	60	4	5	1	4	5	4	2	2	3	3	2	4	4	5	1	3	4	2	1	1	1	3	1	1	5	5	4	2	3
816	1	1	19	6	90	3	4	3	4	4	3	3	3	2	3	4	4	4	4	4	4	5	3	4	4	3	3	3	3	2	3	4	3	3
817	1	1	19	5	60	2	5	3	3	3	2	2	2	2	2	3	3	5	4	3	4	4	4	4	4	4	4	4	4	5	4	4	4	3
818	1	1	19	5	60	1	1	1	1	2	1	2	1	1	1	3	1	4	4	3	4	3	4	4	3	2	4	2	2	3	2	3	4	1
819	2	1	19	1	30	1	2	1	2	1	1	1	1	1	1	3	1	3	4	3	4	5	4	5	5	5	5	4	2	5	5	5	5	3
820	1	1	22	4	120	5	5	4	5	3	5	3	3	4	3	2	4	1	3	2	2	3	1	3	1	3	4	4	5	4	3	3	3	4
821	2	1	20	1	30	2	4	3	2	3	4	4	3	3	3	3	4	2	4	3	4	4	3	5	4	4	4	4	4	4	4	4	4	4
822	2	2	23	1	120	2	2	2	2	2	2	2	2	2	2	2	4	2	2	3	3	3	4	3	4	2	2	3	3	3	2	2	2	2
823	2	1	21	1	180	3	4	4	4	4	3	4	4	3	4	4	3	4	4	4	2	2	3	2	2	2	3	3	4	4	4	4	4	4
824	2	1	19	2	30	3	2	2	2	2	3	4	3	4	3	4	3	4	3	4	2	2	3	3	2	3	2	3	3	3	3	3	3	3
825	2	1	19	1	60	3	5	2	2	5	3	4	2	3	2	4	4	5	5	4	4	5	2	2	3	4	2	3	3	4	3	4	4	4
826	2	1	20	1	15	3	2	2	2	3	2	3	2	2	2	2	3	4	3	2	3	3	2	2	2	2	2	2	3	4	4	3	4	3
827	1	1	19	1	180	2	3	2	3	3	2	2	2	2	2	3	3	2	3	2	3	3	2	2	2	2	2	2	2	3	4	4	4	3
828	2	1	19	2	105	3	3	3	4	4	3	2	3	2	2	2	3	4	4	3	4	4	3	2	3	2	2	1	2	4	4	4	4	3
829	2	1	19	2	30	3	4	2	3	4	2	3	3	3	2	3	3	4	4	3	4	4	2	3	4	3	3	2	3	5	4	5	5	4
830	1	1	19	2	70	5	4	3	3	5	4	3	2	3	2	4	3	2	2	1	3	4	2	2	3	2	4	3	3	5	4	3	2	2
831	1	1	22	1	180	3	3	4	4	3	2	3	3	3	1	1	3	3	3	5	3	1	1	1	2	3	2	1	1	4	2	5	5	2
832	1	2	24	5	120	4	4	4	4	4	5	4	4	3	4	4	5	4	4	5	5	4	4	4	4	3	4	4	5	3	4	4	3	4
833	1	2	20	3	30	1	1	1	1	2	2	1	2	1	2	2	3	3	4	3	3	3	4	3	3	3	4	3	3	3	4	3	2	3
834	1	2	24	4	30	1	2	2	2	2	1	1	1	1	2	1	3	4	4	3	3	4	3	4	4	4	4	2	4	3	1	5	5	3

835	2	2	19	2	90	3	4	2	4	5	4	3	4	4	5	5	5	3	4	4	5	5	4	4	5	5	5	3	5	4	4	4	5	3
836	1	2	21	2	120	4	2	2	3	2	2	2	2	2	2	1	2	2	2	2	2	1	2	2	2	1	1	2	2	4	2	3	4	3
837	1	1	19	4	90	3	2	3	4	3	2	3	3	2	2	3	5	4	3	3	2	4	3	3	3	2	3	4	5	3	3	2	3	4
838	1	1	19	5	60	2	2	3	2	3	2	3	3	2	2	2	3	3	3	4	3	3	4	4	3	4	3	3	3	2	3	2	3	3
839	1	1	19	4	40	5	4	4	5	5	5	4	4	4	4	5	5	4	4	4	5	5	5	4	4	5	5	4	5	5	4	4	5	5
840	1	1	23	2	120	1	1	1	1	1	1	1	1	1	1	1	1	2	1	1	1	1	1	1	1	1	1	1	1	4	1	5	4	1
841	1	1	19	3	120	4	4	4	4	4	4	3	3	3	3	4	4	5	4	4	5	4	4	3	4	4	4	4	4	4	4	4	4	4
842	1	3	24	3	60	1	1	1	1	1	1	1	1	1	1	1	1	4	4	4	4	4	4	4	4	4	4	1	4	4	4	4	4	3
843	1	1	20	3	120	2	2	3	3	3	4	3	4	3	2	3	4	4	4	4	3	3	4	3	4	4	5	3	3	4	4	4	4	4
844	1	1	22	3	120	1	1	1	1	1	1	1	1	1	1	1	1	5	5	5	5	5	5	5	5	5	5	5	5	2	2	2	1	1
845	2	3	19	2	60	2	4	2	2	4	3	3	2	2	2	3	4	4	4	4	4	4	4	4	4	4	4	2	4	3	3	3	4	3
846	2	2	19	2	60	3	3	3	3	2	3	3	3	3	3	2	3	3	3	3	3	3	3	2	3	3	3	3	2	3	3	3	3	3
847	1	1	19	1	60	3	3	3	3	3	3	2	3	3	3	3	3	3	2	2	3	3	3	3	3	3	3	3	3	3	3	3	3	1
848	2	1	19	1	90	2	3	2	2	3	2	2	2	2	2	2	2	3	3	3	3	3	3	2	3	3	3	3	3	3	3	3	2	2
849	2	2	20	1	60	3	3	2	2	2	3	3	2	2	3	2	2	3	3	3	2	3	3	3	3	2	2	3	3	2	2	2	3	2
850	2	1	22	1	60	2	2	2	2	2	1	1	1	1	2	1	1	2	2	2	1	1	1	1	2	1	1	2	1	3	3	3	3	1
851	2	4	20	1	30	1	2	1	3	2	3	2	1	1	1	1	2	4	3	3	4	3	4	4	4	3	4	2	2	1	3	3	2	2
852	1	4	19	5	30	3	4	2	4	4	3	3	3	3	3	3	4	5	4	4	5	5	5	5	5	5	5	4	5	5	5	5	5	5
853	2	1	20	1	30	1	2	2	3	2	2	2	1	1	1	1	2	4	4	4	5	5	5	5	4	4	5	3	3	3	3	2	4	3
854	1	1	23	5	120	3	4	3	4	4	3	3	3	3	4	4	5	4	4	3	3	3	4	4	3	3	4	4	5	3	2	4	4	4
855	1	1	19	3	120	2	4	2	3	4	3	2	2	3	2	3	5	5	4	5	5	5	5	4	4	4	4	5	5	4	3	4	4	3
856	1	1	20	5	120	4	5	4	5	5	4	4	5	4	4	3	4	5	5	3	4	4	4	3	4	4	5	4	4	4	3	4	4	3
857	1	3	22	2	60	3	4	3	5	5	4	5	4	3	4	5	4	5	4	3	4	4	5	4	4	5	4	2	3	4	5	4	3	5
